# *Arabidopsis thaliana* Chromosome 4 Replicates in Two Phases That Correlate with Chromatin State

**DOI:** 10.1371/journal.pgen.1000982

**Published:** 2010-06-10

**Authors:** Tae-Jin Lee, Pete E. Pascuzzi, Sharon B. Settlage, Randall W. Shultz, Milos Tanurdzic, Pablo D. Rabinowicz, Margit Menges, Ping Zheng, Dorrie Main, James A. H. Murray, Bryon Sosinski, George C. Allen, Robert A. Martienssen, Linda Hanley-Bowdoin, Matthew W. Vaughn, William F. Thompson

**Affiliations:** 1 Department of Horticultural Science, North Carolina State University, Raleigh, North Carolina, United States of America; 2 Department of Molecular and Structural Biochemistry, North Carolina State University, Raleigh, North Carolina, United States of America; 3 Departments of Plant Biology, Genetics, and Crop Science, North Carolina State University, Raleigh, North Carolina, United States of America; 4 Cold Spring Harbor Laboratory, Cold Spring Harbor, New York, United States of America; 5 School of Biosciences, Cardiff University, Cardiff, Wales, United Kingdom; 6 Department of Horticulture and Landscape Architecture, Washington State University, Pullman, Washington, United States of America; The University of North Carolina at Chapel Hill, United States of America

## Abstract

DNA replication programs have been studied extensively in yeast and animal systems, where they have been shown to correlate with gene expression and certain epigenetic modifications. Despite the conservation of core DNA replication proteins, little is known about replication programs in plants. We used flow cytometry and tiling microarrays to profile DNA replication of *Arabidopsis thaliana* chromosome 4 (chr4) during early, mid, and late S phase. Replication profiles for early and mid S phase were similar and encompassed the majority of the euchromatin. Late S phase exhibited a distinctly different profile that includes the remaining euchromatin and essentially all of the heterochromatin. Termination zones were consistent between experiments, allowing us to define 163 putative replicons on chr4 that clustered into larger domains of predominately early or late replication. Early-replicating sequences, especially the initiation zones of early replicons, displayed a pattern of epigenetic modifications specifying an open chromatin conformation. Late replicons, and the termination zones of early replicons, showed an opposite pattern. Histone H3 acetylated on lysine 56 (H3K56ac) was enriched in early replicons, as well as the initiation zones of both early and late replicons. H3K56ac was also associated with expressed genes, but this effect was local whereas replication time correlated with H3K56ac over broad regions. The similarity of the replication profiles for early and mid S phase cells indicates that replication origin activation in euchromatin is stochastic. Replicon organization in *Arabidopsis* is strongly influenced by epigenetic modifications to histones and DNA. The domain organization of *Arabidopsis* is more similar to that in *Drosophila* than that in mammals, which may reflect genome size and complexity. The distinct patterns of association of H3K56ac with gene expression and early replication provide evidence that H3K56ac may be associated with initiation zones and replication origins.

## Author Summary

During growth and development, all plants and animals must replicate their DNA. This process is regulated to ensure that all sequences are completely and accurately replicated and is limited to S phase of the cell cycle. In the cell, DNA is packaged with histone proteins into chromatin, and both DNA and histones are subject to epigenetic modifications that affect chromatin state. Euchromatin and heterochromatin are chromatin states marked by epigenetic modifications specifying open and closed conformations, respectively. Using the model plant *Arabidopsis thaliana*, we show that the time at which a DNA sequence replicates is influenced by the epigenetic modifications to the surrounding chromatin. DNA replication occurs in two phases, with euchromatin replicating in early and mid S phase and heterochromatin replicating late. DNA replication time has been linked to gene expression in other organisms, and this is also true in *Arabidopsis* because more genes are active in euchromatin when compared to heterochromatin. The earliest replicating DNA sequences are associated with acetylation of histone H3 on lysine 56 (H3K56ac). H3K56ac is also abundant in active genes, but the patterns of association of H3K56ac with gene expression and DNA replication are distinct, suggesting that H3K56ac is independently linked to both processes.

## Introduction

DNA replication is a fundamental process required for the growth and development of all eukaryotes. This process is regulated both spatially and temporally so that all DNA sequences are replicated exactly once during S phase, insuring that each daughter cell receives a complete copy of the genome. DNA replication initiates from discrete locations on chromosomes known as replication origins (origins) where proteins required for DNA synthesis are recruited by the origin recognition complex (ORC). Once initiated, DNA replication proceeds by elongation to regions where opposing replication forks converge (termination zones). This organization of DNA sequences into regions of initiation, elongation and termination define a replicon – a segment of DNA replicated as a unit by replication forks originating from a single origin [1]–[5]. The time of replication for any particular DNA sequence within a replicon is determined by three factors: its proximity to an origin, the efficiency of initiation at that origin, and the rate of DNA elongation in that region.

The pattern of DNA replication has been determined for multiple eukaryotic genomes ranging from the compact genome of budding yeast to the moderately sized genome of *Drosophila melanogaster* and the large human and mouse genomes [6]–[14]. In budding yeast, DNA sequences acting as origins have a conserved consensus motif, and origin activation appears to follow a strict temporal program [6]. However, recent single molecule studies of DNA replication in yeast [15], [16] suggest that the temporal program likely represents the average replication program for a population of cells, with considerable variation in the order of origin activation in individual cells [17]–[20]. In higher eukaryotes, no consensus sequence for origin DNA has been identified, and some known origins are organized as broad initiation zones containing multiple potential origins [2]–[4]. It is unclear whether origin activation follows a temporal sequence in higher eukaryotes, but origin activation in *Drosophila* is most prevalent in early and late S phase, suggesting some degree of temporal regulation [14]. In mammals, clusters of replicons frequently display coordinate origin activation and are organized into larger replication domains [1], [5], [21]. The organization of replication domains appears to be cell type specific, as differentiation of embryonic stem cell lines to neural precursor cells resulted in the widespread reorganization of replication domains [13]. Differences in replication patterns between cell types have been linked to changes in gene expression and epigenetic modifications [13], [14].

The relationship between gene expression and replication time has been examined in yeast, *Drosophila*, mouse and human cells. In budding yeast, there is little correlation between replication time and gene expression [6]. In higher eukaryotes with more complex genomes, there is a positive correlation between early replication and gene expression, and this correlation is strongest when integrated over large chromosomal domains [7], [8], [10]–[14], [22], [23]. The fact that an open chromatin conformation is necessary but not sufficient for both DNA replication and gene expression may underlie the correlation between these processes [2]–[4], [11], [21], [24].

In general, euchromatin replicates early in S phase and heterochromatin replicates late, although specific types of heterochromatin replicate in early S phase in yeast [6], [8], [21], [25], [26]. Chromatin is subject to a plethora of epigenetic modifications including histone methylation, histone acetylation and DNA cytosine methylation (5mC). The combinatorial effect of these modifications, as well as the association of other chromatin-binding proteins, determines whether DNA adopts a heterochromatic or euchromatic conformation [27]–[29]. Epigenetic modifications associated with heterochromatin and characteristic of silenced genes and transposable elements include tri- and dimethylation of histone H3 lysine 9 (H3K9me), hypoacetylation of histones, and abundant 5mC [27]–[34]. There are conflicting reports for the correlation between heterochromatic marks and late replication, which is surprising given the tight relationship between late replication and heterochromatin [12], [13], [35]. Modifications associated with euchromatin and active or potentially active genes include tri-, di- and monomethylation of histone H3 lysine 4 (H3K4me), hyperacetylation of histones, and 5mC localized to gene coding sequences [27]–[30], [32]–[34], [36]–[38]. Several replication timing studies showed a positive correlation of early replication with H3K4me [12], [13], [35], which may be indirect because H3K4me is associated almost exclusively with genes and gene-rich regions which tend to replicate early [7], [8], [11].

Several lines of evidence suggest that the link between histone acetylation and replication time is more direct. Hyperacetylaton of histone H3 on lysines 9 and 14 (H3K9/14ac) associates with origins in human cells [39]. Hyperacetylation of histone H3 lysine 56 (H3K56ac) associates with early firing origins in budding yeast [40]. Hyperacetylation of histone H4 lysine 16 (H4K16ac) associates with early replicating regions in *Drosophila* cells [14]. In addition, late-firing origins in budding yeast are regulated by a histone deacetylase complex [41]. These and other experiments suggest that histone acetylation may be the best epigenetic determinant of replication time [24], [42]–[44].

Very little is known about the regulation of DNA replication in plants [45]. The core proteins required for DNA replication are conserved between yeast, plants and animals [46], [47]. The replication machinery of plants is more similar to animals than yeast, but many of the genes encoding these proteins have multiple homologs in *Arabidopsis thaliana* suggesting that functional diversification has occurred [47]. DNA fiber autoradiography studies revealed that *Arabidopsis* possesses two families of replicons, one initiating replication early and the other later in S phase [48]. These likely correspond to euchromatic and heterochromatic replicons because, like most eukaryotes, plants replicate heterochromatin later than euchromatin [25].

In contrast, knowledge of epigenetic modifications in *Arabidopsis* has kept pace with other systems, and with few exceptions, these modifications are functionally conserved between plants and animals [28], [29], [49]. The relationship between epigenetic modifications and DNA replication in plants is virtually unexplored. However, DNA replication is required to maintain the repressed state of a negative regulator of flowering in *Arabidopsis* [50], suggesting that the interplay of these processes is crucial for plant growth and development. Similar to the replication machinery, the genes encoding DNA and histone modifying enzymes often have multiple homologs in plants [51]–[53].

*Arabidopsis* with its small, well-characterized genome is an excellent model system for examining the global relationship between DNA replication and chromatin state in higher eukaryotes. The genome of *Arabidopsis* is gene-dense in comparison to mammalian genomes, with roughly the same number of genes encoded by a genome one-twentieth the size [54]–[56]. The genome size of *Drosophila* is similar but encodes half the number of genes [57]. This characteristic of *Arabidopsis* may provide insight into the influence of gene density on DNA replication. In addition, analysis of *Arabidopsis* DNA replication has the potential to uncover features that are unique to plants. The diversification of genes encoding replication-associated proteins and chromatin modifiers suggests that plants may have developed unique mechanisms to regulate DNA replication and to establish and maintain chromatin states. These mechanisms may be related to developmental pathways that are common in plants but rare in other systems. For example, endoreduplication plays a prominent role in plant development and totipotency of plant cells is not limited to germline or embryonic cells. We used a combination of fluoresence-activated cell sorting (FACS) and genomic tiling arrays to profile DNA replication of *Arabidopsis* chr4 in early, mid and late S phase cells. We investigated the relationship between DNA replication, gene expression and chromatin state in analyses of our data and the extensive genomic data available for *Arabidopsis* chr4.

## Results

### S phase replication profiles

**Figure 1:**
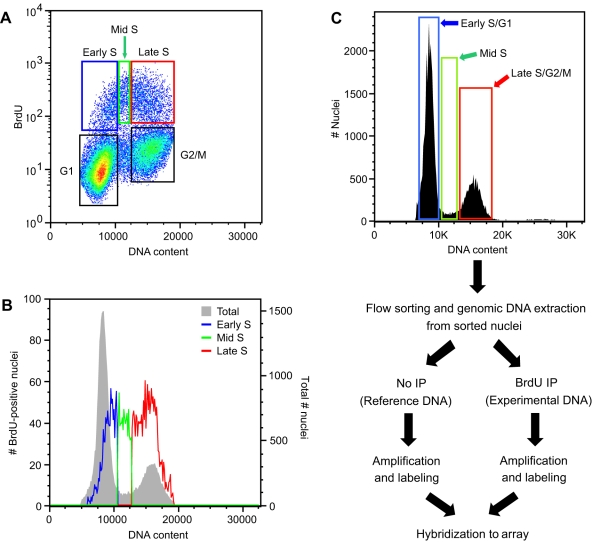
Flow cytometric analysis of *Arabidopsis* cell suspension culture. (A) Analytical FACS profile showing BrdU incorporation into *Arabidopsis* nuclei as a function of DNA content. BrdU incorporation and DNA content of nuclei were visualized using anti-BrdU Alexa 488 conjugate and propidium iodide, respectively. Five boxes are shown, representing nuclei in G1, early S, mid S, late S, and G2/M, respectively. (B) Histogram plots for total and BrdU-positive nuclei from the BrdU labeled cells shown in (A). (C) Flow diagram of FACS-based microarray experiments for profiling replication in early, mid and late S. Cells were pulse-labeled with BrdU, and nuclei isolated. Populations of nuclei in early S/G1, mid S, and late S/G2 were sorted based on DNA content. Genomic DNA was prepared from the sorted nuclei in each fraction and sheared to an average size of 500 bp before heat denaturation and immunoprecipitation with antibodies against BrdU. DNA containing BrdU was amplified, labeled with Cy dyes and hybridized to a tiling array for *Arabidopsis* chr 4.

We used an established *Arabidopsis* Col-0 suspension cell line for the analysis of replication time and optimized the culture conditions to provide ample nuclei from replicating cells for fractionation by FACS (Figure S1 and Text S1). This cell line was also used in recent studies that examined the effects of cell culture on specific epigenetic modifications [34]. We first characterized the relationship between DNA content and replication in this cell line by monitoring the incorporation of the nucleotide analog bromodeoxyuridine (BrdU). An asynchronous population of cells was labeled with BrdU for 1 hour and fixed. Nuclei were isolated, stained with propidium iodide, labeled with a fluorescent anti-BrdU antibody, and analyzed by FACS for DNA content and BrdU incorporation. Nuclei in S phase that incorporated BrdU appeared as a distinct “arc” above the population of cells in G1 and G2/M (Figure 1A). Surprisingly, almost 30% of the S phase nuclei fractionated above the G1 peak, and we designated this population early S phase (Figure 1A and Table S1). Similarly, we designated the 50% of the S phase nuclei that fractionated above the G2/M peak as late S phase. The remaining 20% of S phase nuclei between the G1 and G2/M peaks were designated mid S phase. We estimated the DNA content of the early, mid and late S phase populations at 1.16, 1.49 and 1.95C, respectively (Figure 1B). This distribution of S phase nuclei and DNA content indicated that to get a complete picture of DNA replication during S phase we needed to analyze DNA replication in nuclei that co-sorted with G1 (early S phase) and G2/M (late S phase) peaks.

We profiled DNA replication independently in early, mid and late S phase. We could not sort the early, mid and late S phase nuclei based on BrdU content because visualization of the BrdU degrades DNA. Instead, nuclei were sorted based on DNA content, and BrdU-labeled DNA was separated by immunoprecipitation (Figure 1C). Nuclei in the early S/G1, mid S and late S/G2/M sorts contained different fractions of nuclei in S phase, with the early S/G1, mid S and late S/G2/M sorts containing 4.2, 42.3 and 18.3% BrdU-positive nuclei, respectively (Figure 1A and Table S1). Because of these differences, it was necessary to account for cross contamination associated with sorting (Figure S2A), especially contamination of mid S phase nuclei into the early S/G1 sort (Table S2 and Figure S2B). When corrected for the percentage of nuclei in S phase, we determined that the early, mid and late S phase purity was 69, 94 and 85% respectively (Table S2). In the worst case, 28% of S phase nuclei in the early S/G1 sort were actually in mid S phase (Table S2 and Figure S2B). However, this contaminating population had a DNA content from the lower tail of the mid S phase distribution (Figure S2B).

BrdU-labeled DNA from early, mid or late S phase nuclei was hybridized separately to a tiling microarray that covers 99% of the sequenced regions of chr4 of *Arabidopsis thaliana* with 22,761 PCR-generated probes averaging 1 kb in length [33]. This array was used previously to profile specific epigenetic modifications in this cell line [34]. Microarray results were confirmed by qPCR analysis of 14 selected regions (Tables S3 and S4, Figure S3 and Text S1).

**Figure 2:**
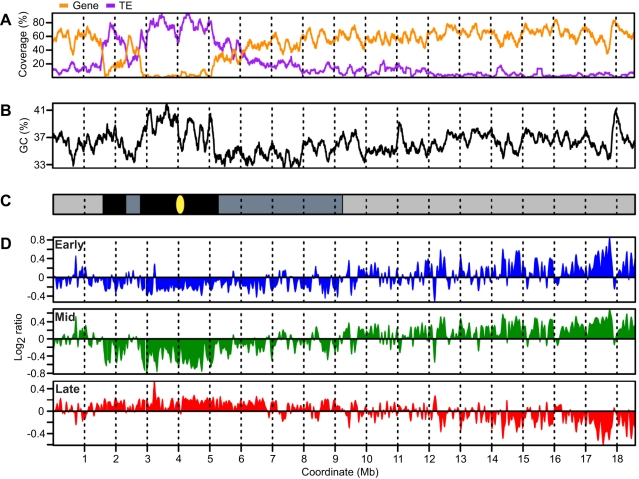
*Arabidopsis* chromosome 4 with replication profiles for early, mid, and late S phase suspension culture cells. (A) Gene and TE coverage were determined using TAIR8 annotation and are expressed as percentage of bases in 1 kb non-overlapping segments occurring in genes or TEs respectively. Overlapping genes or TEs were merged to prevent coverage values exceeding 100%. Data were loess-smoothed using a 150 kb window. (B) GC percentage calculated in 1 kb non-overlapping windows and loess-smoothed using a 150 kb window. (C) Schematic representation of chromosome 4 omitting the telomeres and nucleolar organizing region. The gene-rich euchromatic distal short and distal long arms are shaded light gray while the heterochromatic knob and pericentromere are rich in TEs and are shaded black. The proximal portions of both the short and long arms have intermediate characteristics and are shaded dark gray. (D) Replication profiles for early, mid, and late S phase cells. Replication is expressed as log_2_ ratio of BrdU-labeled sequences in early, mid or late S phase cells with respect to total DNA from the same cells. Data have been normalized and scaled within experiments, but no normalization was performed between experiments.

Figure 2 shows a schematic representation of chr4 including plots for gene and transposable element (TE) coverage (Figure 2A) and GC content (Figure 2B). Chr4 is unusual in that it has three regions of constitutive heterochromatin – the nucleolar organizing region (NOR) at the end of the short arm (not shown), a 700 kb heterochromatic knob centered at 2 Mb, and 2.5 Mb of pericentromeric heterochromatin centered at 4 Mb (Figure 2C) [58], [59]. These heterochromatic regions were used as boundaries to subdivide chr4 into six regions for subsequent analyses – the distal short arm, the heterochromatic knob, the proximal short arm, the pericentromere, the proximal long arm and the distal long arm (Figure 2C). The boundaries of most of these regions are evident from gene and transposable element (TE) coverage and to some extent from the GC content profile (Figure 2A and 2B). The boundary between the proximal and distal long arms is less evident and was chosen based on the replication time results presented below.

The replication profiles were generated from the microarray data by applying a loess algorithm in a 150-kb window to smooth the probe-level data (Figure 2D). The early and late profiles display remarkable complementarity (R = −0.83), i.e. regions of chr4 enriched for BrdU in early S phase cells are depleted in late S phase cells. Early replication is most prevalent in the distal long arm, a euchromatic region rich in genes with few TEs. Late replication predominates in the heterochromatic knob and pericentromere of chr4, but regions of late replication are also dispersed in other parts of chr4, most notably the proximal long and short arms.

The replication profiles for early and mid S phase cells are surprisingly similar (R = 0.87) (Figure 2D). The most evident difference is a broadening and merging of early replicating regions in the mid S phase profile. The DNA replicating in mid S phase represents nearly the same population of sequences as that replicating in early S phase even though FACS analysis demonstrates that the early and mid S phase nuclei have notably different DNA content (Figure 1, Figure S2 and Tables S1 and S2). Like early S phase, the mid S phase profile is distinct from the late profile (R = −0.85). The similarity of the early and mid S phase profiles is not consistent with a fixed order of origin activation and, instead, suggests that origin activation in early and mid S phase is stochastic. Together, the early, mid and late S phase profiles suggest that DNA replication in *Arabidopsis* cells is biphasic, a result consistent with a previous report that *Arabidopsis* DNA replication takes place in two distinct stages [48].

### Segmentation of replication profiles

**Figure 3:**
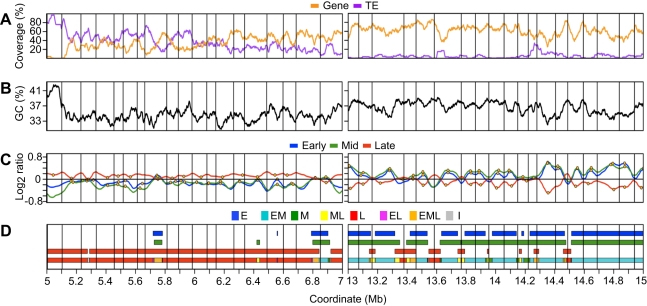
Analysis of replication profiles for segments of coordinate replication time and identification of initiation and termination zones. Representative late- (coordinates 5–7 Mb) and early-replicating (coordinates 13–15 Mb) regions of chromosome 4 are shown. (A) Gene coverage and TE coverage as in Figure 2 but the window for loess-smoothing is 50 kb. Vertical gridlines across all panels are described in (C). (B) GC percentage loess-smoothed using a 50 kb window. (C) Replication profiles as in Figure 2 for early, mid, and late S phase cells. Putative initiation zones are shown as orange circles while putative termination zones are shown as vertical gridlines. (D) Segmentation of replication profiles into segments of coordinate timing. Segments of early, mid, or late replication timing were identified within each experiment as regions where the loess-smoothed profile showed enrichment for BrdU-labeling (log_2_ ratio >0). A final step was performed to reconcile timing between experiments and to account for the overlap of segments between experiments, as shown in the composite model (lowest line). Segments were classified as E (only early), EM (early and mid), M (only mid), ML (mid and late), L (only late), EL (early and late), EML (early, mid and late) or I (indeterminate, no enrichment in any experiment).

To facilitate further analyses, we performed a two-step segmentation of the early, mid and late S phase profiles to assign a replication time for each microarray probe. Figure 3 illustrates this process for two chr4 regions representative of early and late replicating regions. In the first step, we identified contiguous segments of probes showing coordinate replication times (log_2_ ratio >0) within each smoothed profile, thereby defining segments of early, mid or late replicating DNA (Figure 3C and 3D). In the next step, we reconciled the replication times between experiments by determining the regions of overlap between the early, mid and late segments (Figure 3D). This analysis identified segments of DNA replicating only in early S phase (E), in both early and mid S phase (EM), only in mid S phase (M), in both mid and late S phase (ML), only in late S phase (L), in early and late S phase (EL), throughout S phase (EML), and segments of indeterminate replication time (I) that did not show enrichment in any experiment (Table S5).

**Figure 4:**
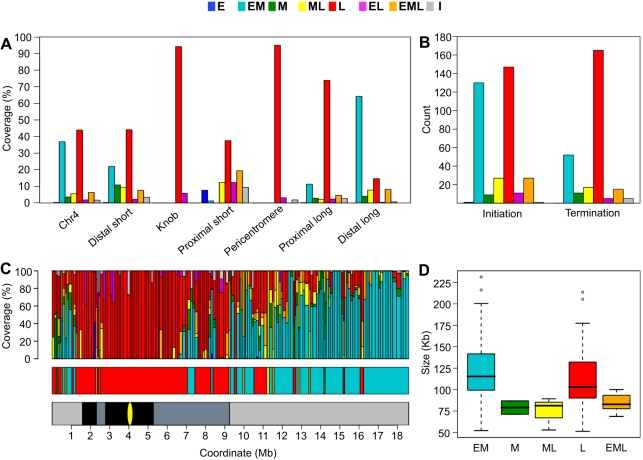
Analysis of chromosome 4 replication timing segments and replicon structure. (A) Distribution of replication timing segments for chr4. The majority of chr4 replicates as either EM (37%) or L segments (44%). The heterochromatic knob and pericentromere replicate almost exclusively as L segments while EM segments dominate the gene-rich distal long arm. The distal short, proximal short and proximal long arms display complex replication timing patterns intermediate between the heterochromatic and euchromatic regions. (B) Distribution and timing of replication initiation and termination zones. Replication timing was assigned to all zones from the timing of overlapping probes. The occurrence of initiation zones in EM and L segments is proportional to the coverage of EM and L segments. In contrast, termination zones are overrepresented in L segments. (C) Schematic representation of replicons, replication timing, and replication domains for chr4. In the top panel, each vertical bar represents a replicon with the width of the bar proportional to the length of the replicon. Subdivisions within the bar indicate the percentage of probes within the replicon that replicate in a given time window. The middle panel illustrates the clustering of replicons with similar timing into replication domains. For reference, the lower panel shows the chromosomal zones defined in Figure 4C. (D) Analysis of replicon size as a function of replication timing. 70 EM, 2 M, 3 ML, 80 L and 8 EML replicons were identified for chr4. Lengths of identified replicons are shown as a boxplot with whiskers extending to 1.5 times the interquartile range. Outliers are shown as open cirlces. The differences in size between replicon classes were not statistically significant as determined by a t-test.

The majority of chr4 replicates as either EM (37%) or L (44%) when segment length is taken into account (Figure 4A). Only 4% of chr4 replicates exclusively in mid S phase (M), while 6% replicates as ML and 6% replicates as EML. The positions of these segment types with respect to EM and L segments suggest that many of the M, ML and EML segments are regions of DNA elongation between EM segments or transition zones between early to late replication (Figure 3). In regions of predominately late replication, M, ML and EML segments are often located between larger flanking L segments (Figure 3), suggesting that they contain the DNA replication origins for the flanking regions. The EL segments comprise only 2% of chr4 and are enriched for repetitive sequences (Table S6). Thus, at least some EL segments are likely to be artifacts created by cross hybridization on the microarray. I segments, which comprise 2% of chr4, also have an elevated repeat content (Table S6). Another possible explanation for EL and I segments is that replication time in these regions is driven by allele-specific gene expression and/or epigenetic modifications (see below) [12], [60].

We also determined the replication times of the six chr4 regions defined in Figure 2. The heterochromatic knob and pericentromere replicate almost exclusively as L segments while the gene-rich distal long arm replicates predominately as EM segments (Figure 4A). The replication time of the distal short and proximal short regions is more complex, perhaps influenced by the flanking heterochromatic regions (Figure 2). The proximal long arm displays a surprising amount of late replication despite the fact that this region is not constitutive heterochromatin [59], although it does have lower gene and higher TE content than the distal long region (Figure 2).

### Identification of initiation and termination zones and replicon boundaries

Within a given replicon, the DNA closest to the origin will replicate earliest while the DNA located at termination zones, regions where opposing replication forks converge, will replicate latest. Replication time profiles have been used to identify both initiation and termination zones [6], [7], [14]. Initiation zones manifested as local maxima in the early and mid S phase profiles and as local minima in the late S phase profile (Figure 3C). Conversely, termination zones manifested as local minima in the early and mid S phase profiles and as local maxima in the late S phase profile (Figure 3C). We identified initiation and termination zones by computationally determining probes occurring at local maxima and minima in the loess smoothed profiles. We did not treat individual probes as initiation or termination zones and, instead, defined zones as 10 kb segments centered at the identified probes. Any zones that overlapped were then merged into a single zone. Replication time for each zone was determined from constituent probes.

The number of initiation and termination zones was consistent between experiments (Table S7 and Figure 3C). However, their positions were more consistent between the early and mid S phase profiles versus comparisons with the late S phase profiles, e.g. 80% of the initiation zones identified in the early S phase profiles are within 20 kb of an initiation zone identified in the mid S phase profiles while this figure dropped to 65% when comparing the early and late S phase initiation zones (Table S8). This difference is not unexpected given that initiation zones are more likely to replicate in early or mid S phase while termination zones are more likely to replicate in late S phase.

We then examined the frequency of initiation and termination zones as a function of replication time. In *Drosophila*, initiation sites are more abundant in late replicating DNA than in early replicating DNA with very little initiation occurring in mid S phase [14]. In *Arabidopsis*, we found that the distribution of replication times for the initiation zones reflected the distribution of replication times for chr4 with initiation zones prominent only in EM (37%) and L (42%) segments (compare Figure 4A and 4B). Thus, unlike *Drosophila*, initiation sites are no more abundant in late than in early replicating DNA. However, *Arabidopsis* appears similar to *Drosophila* [14] in that the majority of termination zones are located in L (61%) rather than EM (19%) segments (Figure 4B). These results indicate that DNA replication in late S phase includes elongation from origins that have fired earlier in S phase as well as initiation and elongation from late firing origins.

Higher eukaryotes do not possess replicons in the strictest sense of the term, but rather the concept of a “relaxed replicon” likely applies [2]–[4]. In this model, replication origins are not rigidly defined, and replicon boundaries can vary from cell to cell. We defined the boundaries of these relaxed replicons (hereafter referred to as replicons) using a subset of the termination zones. Where possible, we used termination zones that were identified in early, mid and late S phase cells. Where termination zones differed between experiments, we preferentially used termination zones enriched in late S phase cells or local minima from early or mid S phase cells for EM-replicating segments (Figure 3C and Table S7 and Materials and Methods). In this way, we identified 164 termination zones that defined 163 putative replicons across chr4 with a median length of 107 kb. This replicon size is consistent with previous measurements of single replicons in *Arabidopsis* [45], [48], although we cannot exclude the possibility that at least some of these replicons are clusters of smaller replicons. The majority (154) have at least one putative initiation zone (Figure 3C and Table S9). This strategy worked well for the euchromatic regions of chr4, particularly the distal long and distal short arms, where the predicted termination zones were consistent between early, mid and late S phase cells (Figure 3C). There was less agreement between profiles for the other chr4 regions, and replicon boundaries in the late-replicating regions are defined primarily from the late S phase profiles (Figure 3C and Table S7).

The assignment of a specific replication time to individual replicons is complex because a replicon can be comprised of DNA segments with replication times that cover the entirety of S phase. To simplify the analysis, we classified replicons based on the replication time of the probes comprising the greatest proportion of a replicon, e.g. a replicon comprised of 45% EM probes, 40% L probes and 15% M probes would be classified as EM. Figure 4C (top panel) shows a schematic representation of chr4 replicons with the replication times for the constituent probes. The complexity of replication time within replicons likely reflects several factors including time and efficiency of origin firing, the number of origins within initiation zones, and the rate of elongation by DNA polymerase in specific contexts [2]–[4], [24].

In *Drosophila*, the interval between termination zones varies between early S phase and late S phase, with increased initiation in late S phase resulting in more closely spaced termination zones [14]. The size of the *Arabidopsis* replicons does not vary significantly between EM and L replicons (Figure 4D). While M, ML and EML replicons are smaller than either EM or L replicons, the difference in size is not statistically significant (Figure 4D). The similar size of EM and L replicons follows from the previous observation that initiation zones are no more abundant in late replicating regions than in earlier replicating regions (Figure 4B).

In mouse cells, replicons are organized into replication domains consisting of large clusters of replicons with similar replication times [13], [22]. In *Drosophila* cells, clustering is less evident with replication profiles showing distinct peaks of early replication [14]. *Arabidopsis* appears more similar to *Drosophila* in this regard, but the 163 chr4 replicons could be organized into 41 replication domains based on their replication time (Figure 4C, middle panel, and Table S10). There are a few large replication domains, including a 4.5 Mb L domain (coordinates 2.6–7.1 Mb) that encompasses the entire pericentromere and portions of the proximal short and long arms, and a 2.3 Mb EM domain (coordinates 16.2–18.5 Mb) in the distal long arm (Figure 4C, middle panel). However, the mean length of chr4 replication domains is 450 kb which is considerably smaller than the 1 Mb reported for mouse cells [13]. This difference in replicon organization may be related to genome size. The genome sizes of *Arabidopsis* and *Drosophila* are similar at 115 and 122 Mb, respectively [54], [57], while the mouse genome is estimated at 2500 Mb [56].

### Genetic and epigenetic features and replication time

Replication time has been correlated with both genetic and epigenetic features in other model systems [7], [8], [11]–[14]. The replication profiles (Figure 2) show that on the scale of the entire chromosome, EM replication is associated with euchromatic regions while L replication is associated with heterochromatic regions. To examine the relationships between replication time and both genetic and epigenetic features in more detail, we generated a database for computational analysis that incorporates our replication time data, the *Arabidopsis* TAIR 8 genome annotation [61], and epigenetic information for the *Arabidopsis* cell line [34]. We performed our analyses both on the level of individual probes and within the context of replicons.

To compare the genetic and epigenetic features of probes with different replication times, we partitioned the data into six smaller data sets based on the chr4 regions (Figure 2). This approach was necessary because heterochromatin replicates almost exclusively late, so any analysis that does not account for this fact merely compares heterochromatin to euchromatin. We then used a series of one-sample statistical tests to query whether probes with specific replication times were enriched or depleted for a specified genetic or epigenetic feature relative to the mean for that feature within a given region. This analysis is equivalent to comparing replication segments, but has the advantage of controlling for segment length by using probe numbers. Results for the proximal and distal portions of long arm are presented in Table 1. (The complete analysis is in Table S11.)

**Table 1:** Probe-level analysis of replication timing, genetic and epigenetic features.

Region	Replication time^1^	Probe Count	GC content (%)^2^	Gene coverage (%)^2^	TE coverage (%)^2^	H3K4me1/2 (%)^3^	H3K9me2 (%)^3^	H3K56ac (%)^3^	5mC (%)^3^
Proximal Long									
	All	4794	35.1	47.9	13.5	37.8	32.3	35.5	43.0
	EM	519	34.2***	44.5	10.8	26.9***	20.0***	50.0***	28.9***
	M	134	34.6	54.2	12.1	37.8	18.8*	41.2	29.5**
	ML	99	34.8	52.9	16.4	48.5	8.8***	33.3	28.8*
	L	3577	35.3	47.9	13.6	39.1	34.7*	32.2***	46.6***
	EL	108	34.7	52.3	11.5	42.0	39.6	50.0*	40.7
	EML	214	34.3	48.9	16.8	38.1	27.0	47.3**	35.7
	I	133	35.4	45.0	16.3	26.5*	23.3	44.8	30.4*
Distal Long									
	All	10484	36.6	62.8	3.8	36.3	7.0	37.9	25.6
	EM	6693	36.2***	60.1***	3.6	32.6***	5.3***	41.0***	21.5***
	M	427	37.6***	65.9	4.5	39.4	4.2	36.4	22.4
	ML	832	37.4***	69.0***	4.1	43.3***	7.1	30.5***	30.9***
	L	1568	38.0***	72.7***	3.4	48.3***	14.0***	28.7***	40.1***
	EL	39	34.8	58.7	10.0	17.1**	4.5	68.8***	16.2
	EML	853	36.1**	59.3*	4.3	34.1	5.2	37.3	25.9
	I	69	37.6	55.5	9.1	25.0	29.6***	32.8	30.8

**1** E probes were omitted from the table because n<20 for both regions.

**2** Probe GC content, gene coverage and TE coverage were calculated and compared to the mean for all probes in that region using one-sample t-tests. Means that were significantly different from the mean for all probes in that region are indicated with *P* value significance codes as follows: * *p*<0.05, ** *p*<0.01, *** *p*<0.001.

**3** Probes positive for the indicated epigenetic modifications were tabulated and compared to the percentage for all probes in that region. Percentages signficantly different than the percentage for all probes in that region are indicated. *P* values were calculated from the binomial distribution with significance codes as above.

In animal systems, early replication positively correlates with gene and GC content when integrated over large domains [7], [12], [13], [62]. We found that the GC content of EM probes is depleted relative to the distal long arm, whereas the L probes are GC-enriched. EML probes have a GC content similar to EM probes, but M and ML probes are also GC-enriched. These results are likely linked to the gene coverage of these probes, with EM and EML probes showing depleted gene coverage and M, ML and L probes showing enriched gene coverage. The sequence content of the proximal long arm is different from the distal long arm, showing both a lower GC and gene content. However, the EM probes still show a lower GC content relative to the entire region. This depletion of gene and GC content in early-replicating regions contrasts with mammalian systems, and may reflect differences in genome structure.

In both animals and plants, H3K4me is almost exclusively genic and correlates with gene expression [27], [29], [30], [32], [34], [36], [37], with H3K4me3 having the strongest positive effect on gene expression in *Arabidopsis* [37]. H3K4me3 has been linked to early replication in mouse cells [13], and all forms of H3K4me correlate with early replication in human cells [12], [35]. We found that H3K4me1/2 is depleted in EM probes and enriched in ML and L probes in the distal long arm, consistent with the gene coverage. Despite its lower gene coverage, the proximal long arm has an abundance of H3K4me1/2 similar to that of the distal long arm, due in part to the gain of H3K4me1/2 by certain classes of TEs [34]. While we detected a depletion of H3K4me1/2 in EM probes, we did not detect a significant enrichment of H3K4me1/2 in L probes relative the proximal long arm as a whole.

DNA cytosine methylation (5mC) is found in the coding region of genes in the euchromatic regions of *Arabidopsis*, often in conjunction with H3K4me1 [33], [34], [37], [38], [63]. Like H3K4me1/2, 5mC is depleted in EM probes and enriched in ML and L probes in the distal long arm. The distribution of 5mC differs between the proximal long arm and the distal long arm. While 88% of 5mC is genic in the distal long arm, the percentage drops to 60% in the proximal long arm, and much of the 5mC in this region is associated with TEs and other repetitive sequences located in heterochromatin [33], [34]. We found a depletion of 5mC in EM, M and ML probes and an enrichment in L probes, which likely reflects the heterochromatic character of L probes in the proximal long arm.

To confirm this hypothesis, we examined the distribution of histone H3K9me2, which is associated with heterochromatin in *Arabidopsis* [31], [34], [64]. While H3K9me2 is not an abundant feature in the distal long arm, it is depleted in EM and M probes and enriched in L probes in this region, suggesting that some L probes are located in cryptic or facultative heterochromatin [28], [31], [65]. H3K9me2 is much more abundant in the proximal long arm, principally due to the elevated TE and repeat content of this region [33], [34], [58]. Again, H3K9me2 is depleted in EM, M and ML probes and enriched in L probes. The abundance of H3K9me2, 5mC and late replication in the proximal long arm suggests that much of this region should be considered cryptic or facultative heterochromatin.

Finally, we examined the correlation between H3K56ac and replication time. H3K56ac is associated with multiple biological processes that require an open chromatin conformation, including DNA replication, repair and transcription [40], [66]–[71]. H3K56ac is enriched in gene promoter regions in *Arabidopsis* suggesting a role in transcription [34]. In both the distal and proximal long arms, we detected enrichment of H3K56ac in EM probes and depletion in L probes. H3K56ac is also enriched in EL probes in both the proximal and distal long arms and in EML probes in the proximal long arm. The enrichment of H3K56ac in regions depleted for genes and the epigenetic marks associated with genes raises the possibility that some of the H3K56ac detected in our cells may be related to DNA replication rather than gene transcription.

### Distribution of genetic features and epigenetic modifications within replicons

To explore the relationship between genetic and epigenetic features and replication time in more detail, we performed further analyses in the context of the replicons identified above, again restricting our analysis to the long arm of chr4. We compared the overall content of genetic features and epigenetic modifications between EM and L replicons. We found that gene coverage/content, GC content and H3K56ac are higher in EM than in L replicons, whereas L replicons are enriched for TEs, H3K9me2 and DNA 5mC (Table 2). H3K4me1/2 is similar in EM and L replicons (Table 2). While these results are more consistent with animal systems, the results for gene coverage, GC content and H3K4me1/2 seem to conflict with the probe-level analysis presented above. However, two factors must be considered. First, 62 of the 66 EM replicons are located in the distal long arm while 31 of the 42 L replicons are located in the proximal long arm, and the distal long arm has a higher gene content and GC content than the proximal long arm (Table 1). Second, many of the EM and L replicons are comprised of DNA segments that replicate in various parts of S phase, e.g. the termination zones of EM replicons often replicate in late S phase (Figure 3 and Figure 4C). Thus, integration of genetic and epigenetic features over large regions such as replicons may obscure finer relationships.

**Table 2:** Analysis of genetic and epigenetic features for EM and L replicons for the long arm of chr4.

Feature^1^	EM mean	L mean	t^2^	df^2^	p-value^2^
GC content (%)	36.4	35.7	−3.0227	79.665	3.369E-03
Gene coverage (%)	61.5	52.7	3.2884	58.609	1.707E-03
Gene content (genes/Mb)	319	256	4.6938	85.945	1.005E-05
TE coverage (%)	4.14	11.5	−6.4073	51.927	4.337E-08
TE content (TEs/Mb)	114	344	−7.039	49.125	5.669E-09
H3K56ac	39.5	34.2	3.1194	86.584	2.461E-03
H3K4me1/2	35.7	38.6	−1.262	68.43	2.112E-01
H3K9me2	6.6	27.4	−6.2068	44.587	1.594E-07
DNA 5mC	24.5	42.1	−7.1557	56.66	1.804E-09

**1** Values were calculated as in Table 1. Gene and TE content are based on the TAIR8 annotation using the transcription start site for genes and the low coordinate for TEs.

**2** Student's t-test was used for statistical comparisons.

**Figure 5:**
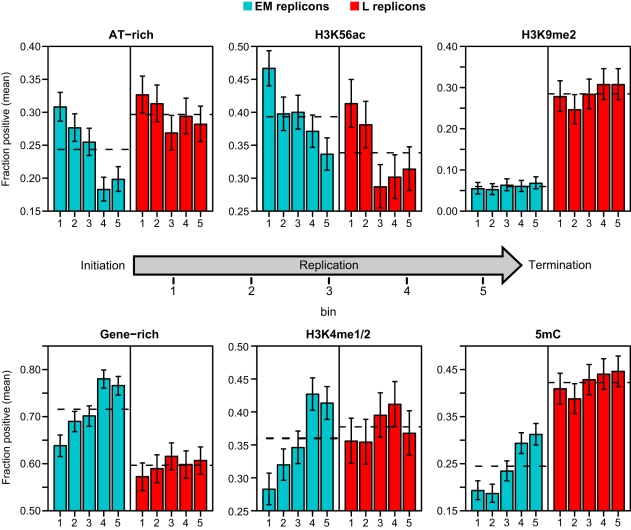
Distribution of genetic and epigenetic features within replicons. The proportion of AT-rich and gene-rich probes and probes positive for H3K56ac, H3K4me1/2, H3K9me3 and 5mC were calculated for each EM or L replicon in 10% intervals and binned according to position in the replicon as described in the text. Bin 1 represents the innermost 20% of the replicon while bin 5 covers the outermost 20% proximal to the termination zones. The barplots show the mean proportion in each bin for EM and L replicons. Error bars indicate the 95% confidence intervals as determined from the binomial distribution. The dashed horizontal lines indicate the mean proportion across the replicons regardless of position.

To further resolve these relationships, we devised an analysis that examined the distribution of features within an “average” replicon. A similar strategy was used to examine the distribution of epigenetic modifications across genes [33], [34]. Each putative replicon in the proximal and distal long arms was divided into 10 intervals, each comprising 10% of its length. Unlike genes that have a definite polarity, most replicons are products of bidirectional fork progression and can be treated as symmetrical [1]–[5]. Hence, we combined our 10 intervals into 5 bins with the two innermost intervals near initiation zones comprising bin 1 and the two outermost intervals near termination zones comprising bin 5. We determined the occurrence of gene-rich, AT-rich, H3K4me1/2, H3K9me2, H3K56ac and 5mC probes within each bin across EM and L replicons separately (Figure 5).

We detected spatial correlations for both genetic and epigenetic features in EM replicons (Figure 5). Both AT-rich (top 25%) and H3K56ac probes are more abundant near initiation zones and depleted near termination zones (Figure 5). In contrast, the distribution of gene-rich (top 25%), H3K4me1/2 and 5mC probes show opposite trends (Figure 5). H3K9me2 is sparse in EM replicons, and there is no spatial correlation (Figure 5). These results suggest that DNA replication initiates in AT-rich intergenic regions with an open chromatin conformation and proceeds by elongation into gene-rich regions where the epigenetic features associated with the gene regulation specify a more complex chromatin structure. Most of the spatial correlations do not apply to L replicons, although there is a clear enrichment of H3K56ac near initiation zones (Figure 5). This analysis reconciles the probe-level (Table 1) and replicon analyses (Table 2), demonstrating that genetic and epigenetic features have both short and long range influences on replication time.

### DNA replication, gene expression, and epigenetic modifications

**Figure 6:**
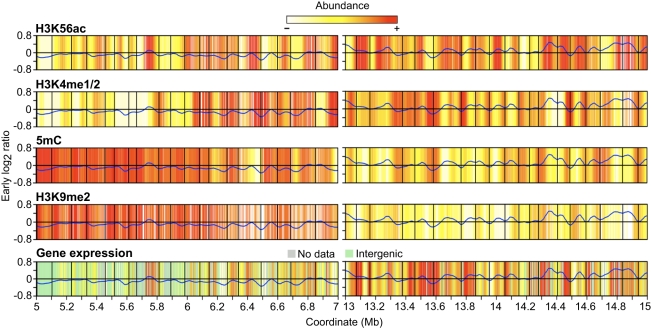
Heat maps of epigenetic modifications and gene expression. Representative late- and early-replicating regions as in Figure 4 are shown. Loess-fitted probe enrichment values for H3K56ac, H3K4me1/2, 5mC and H3K9me2 are presented as heat maps. Gene expression is presented similarly and corresponds to loess-fitted gcRMA expression values with grey regions indicating genes not present on the ATH1 array while green regions indicate intergenic probes. The early S phase replication profile is shown to indicate replication time. H3K56ac is elevated in large regions near initiation zones while H3K4me1/2 and 5mC are enriched in large regions near termination zones, but there are no consistent correlations between gene expression, replication timing, and epigenetic modifications on this scale.

To determine if the increased H3K56ac near initiation zones is linked with gene expression, we looked more closely at the relationship between replication time, gene expression and epigenetic modifications. Previous analysis of these cells showed that H3K56ac is enriched at the 5′ end and promoters of genes, while H3K4me1/2 and 5mC are enriched in the body of genes [34]. To discern broad patterns of epigenetic modification and gene expression, we generated heat maps of the epigenetic data using a loess algorithm as we did for replication time. We determined gene expression in our cells using existing microarray data [34] and used two metrics to measure gene expression. The presence/absence of a transcript was determined using the Affymetrix Micro Array Suite 5.0 algorithm (MAS5) [72]. If the transcript was present, we considered the gene to be active. Gene expression levels were estimated using the gcRMA algorithm [73]. For the heat maps, we mapped the gcRMA expression values to the microarray probes prior to applying the loess algorithm. Representative late and early replicating regions of chr4 are shown in Figure 6. Elevated levels of H3K56ac are frequently associated with regions near replicon initiation zones whereas elevated levels of H3K4me1/2, H3K9me2 and 5mC are often near termination zones. Gene expression showed less clear-cut results sometimes colocalizing with H3K4me1/2 near termination zones and sometimes with H3K56ac near initiation zones.

We then examined the effect of epigenetic modifications on gene expression and replication time at the level of genes. The 2844 chr4 genes with available expression and epigenetic data were classified into 16 groups based on the pattern of all possible combinations of the four epigenetic modifications examined in our cells [34]. Replication time for each gene was derived from the overlapping probes. Using MAS5 presence/absence calls, we estimated that 61% of chr4 genes are active in our cells. Using this as a baseline, we ranked the 16 epigenetic patterns by increasing gene activity, with genes displaying pattern 1 having the highest probability of expression and genes with pattern 16 having the lowest (Table 3). Genes with pattern 1, which constitute the largest group, are positive for H3K4me1/2, H3K56ac and 5mC (Table 3). The presence of H3K4me1/2 and 5mC on expressed genes is consistent with previous studies showing that these marks can potentiate gene expression in *Arabidopsis* [37], [38], [63]. Strikingly, H3K56ac is the only epigenetic modification found in all patterns that show increased gene activity (Table 3). A positive correlation between gene expression and H3K56ac has been shown in other organisms [66], [70], [71], [74], and we show that this correlation exists in *Arabidopsis*. For the remaining patterns, H3K9me2 showed a clear association with reduced gene activity while genes lacking detectable H3K4me1/2, H3K9me2, H3K56ac or 5mC also showed low activity (Table 3).

**Table 3:** Effect of epigenetic modifications on gene expression and replication timing.

Epigenetic modifications^1^				Pattern^2^	Total	Expression Level^3^	Gene activity (%)^4^	E (%)^5^	EL (%)^5^	EM (%)^5^	EML (%)^5^	I (%)^5^	L (%)^5^	M (%)^5^	ML (%)^5^
all genes					2844	5.52	60.6	0.2	1.4	48.5	7.1	1.2	30.4	4.0	7.2
K4	K9	K56	5mC												
+	−	+	+	1	818	6.20	75.8***	0.0	0.9	44.7**	8.1	0.7	33.6**	5.0*	7.0
−	−	+	+	2	112	6.79	74.1**	0.9*	3.6*	46.4	9.8	1.8	25.9	4.5	7.1
−	−	+	−	3	435	6.58	70.6***	0.0	1.2	65.8***	6.9	1.2	14.9***	3.9	6.2
+	−	+	−	4	461	6.00	68.1***	0.0	0.7	55.5***	6.7	0.4	26.0*	3.9	6.7
+	−	−	+	5	168	4.90	52.0*	0.0	1.8	44.1	4.8	1.2	34.5	4.2	9.5
+	+	+	−	6	73	4.00	52.1	0.0	2.7	49.3	2.7	1.4	30.1	4.1	9.6
+	+	+	+	7	160	4.68	50.6*	1.3**	2.5	21.9***	10.6*	1.9	57.5***	0.6*	3.8*
−	+	+	−	8	54	4.53	50.0	3.7**	1.9	55.6	1.9	0.0	27.8	1.9	7.4
−	+	+	+	9	45	4.16	37.8**	0.0	6.7**	40.0	2.2	2.2	40.0	4.4	4.4
+	−	−	−	10	123	3.81	34.2***	0.0	0.0	52.0	6.5	0.8	28.5	2.4	9.8
+	+	−	−	11	15	3.46	33.3*	0.0	0.0	40.0	13.3	0.0	33.3	6.7	6.7
−	−	−	+	12	81	3.98	29.6***	1.2*	1.2	39.5	7.4	2.5	33.3	3.7	11.1
−	−	−	−	13	191	3.99	29.3***	0.0	0.0	55.5*	8.9	2.1	17.3***	5.2	11.0*
+	+	−	+	14	44	3.52	25.0***	0.0	2.3	18.2***	2.3	2.3	70.5***	0.0	4.6
−	+	−	+	15	48	2.87	12.5***	0.0	8.3***	12.5***	4.2	4.2*	66.7***	0.0	4.2
−	+	−	−	16	16	2.53	12.5***	0.0	6.3*	25.0*	0.0	6.3*	50.0*	6.3	6.3

**1** Epigenetic modifications for chr4 genes were determined from the epigenetic modifications of overlapping probes. Genes with no or incomplete epigenetic data were omitted. K4 – H3K4me1/2, K9 – H3K9me2, K56 – H3K56ac and 5mC – DNA cytosine methylation.

**2** Pattern numbers are provided for convenience and correspond to the rank of gene activity.

**3** Mean expression levels were determined with gcRMA and are provided for comparison only.

**4** Gene activity is derived from MAS5 presence/absence calls. The percentage of active genes with the specified epigenetic modifications was compared to the percentage of active genes regardless of modifications. Percentages signficantly different than the percentage for all genes are indicated. *P* values were calculated from the binomial distribution with significance codes as follows: * *p*<0.05, ** *p*<0.01, *** *p*<0.001.

**5** The replication time for all genes was determined from the replication time of overlapping probes. Percentages reflect the number of genes within a group with the indicated replication time. Groups with a significantly higher or lower percentage than the percentage of all genes with that replication time are indicated. *P* values were calculated from the hypergeometric distribution with significance codes as follows: * *p*<0.05, ** *p*<0.01, *** *p*<0.001.

Studies in other model organisms have shown a positive correlation between gene transcription and early replication [7]–[14]. When examined independent of epigenetic modifications, genes are significantly more likely to be expressed if they replicate EM rather than L (Table 4). Of chr4 genes, only genes with patterns 3 and 4 are more likely to replicate EM. Interestingly, genes with patterns 3 and 4 are distinguished from genes with patterns 1 and 2 by the lack of 5mC (Table 3). Despite their high frequency and levels of expression, genes with pattern 1 showed a slight tendency to replicate L and genes with pattern 2 showed no clear bias for either EM or L replication. Genes with patterns 7, 14 and 15 are more likely to replicate L than EM, and each of these patterns is characterized by the presence of H3K9me2 and 5mC (Table 3). In summary, the increased expression of EM-replicating genes is associated with enrichment of this population for genes displaying H3K56ac but lacking 5mC as well as with depletion of genes bearing the repressive combination of H3K9me2 and 5mC.

**Table 4:** Relationship between replication time and gene activity.

Replication timing	Total	Gene activity (%)^1^	Expression Level^1^
All genes	2844	60.55	5.51
E	6	50	5.11
EL	39	61.54	5.29
EM	1379	65.41***	5.82
EML	203	66.5	5.93
I	33	48.48	5.07
L	865	52.49***	4.95
M	113	60.18	5.37
ML	206	58.25	5.67

**1** See Table 3.

Allele-specific differences in replication time have been observed in animals [12], [60]. This can occur when one allele of a gene bears activating epigenetic modifications while the other allele bears repressive modifications, and could give rise to EL, EML or I replication time. Genes with patterns 6 through 9, 11 and 14 bear such modifications, and we did observe a slight enrichment of pattern 9 for EL genes and pattern 7 for EML genes (Table 3). However, the majority of the EL, EML and I segments cannot be explained by allele-specific replication timing. In many cases, genes that replicate EL, EML or I have only activating or repressive marks (Table 3). As stated above, many of these segments are associated with TEs and other repetitive elements.

The heat maps suggested that much of the H3K56ac on chr4 is associated with early replication and not gene expression (Figure 6). To examine this more closely, we determined whether the H3K56ac near the initiation zones of replicons in the long arm of chr4 was due to genes with epigenetic patterns 1 through 4 or reflected H3K56ac in intergenic sequences as well. Genes with pattern 3, positive only for H3K56ac, show a slight enrichment near initiation zones of EM replicons (Figure S4). An analysis of intergenic regions of chr4 revealed that the two most abundant epigenetic patterns are 3 (H3K56ac only) and 13 (no detected modifications) (Table S12). In the long arm of chr4, intergenic regions with pattern 3 are enriched near initiation zones and depleted near termination zones, but intergenic regions with pattern 13 are uniformly distributed across replicons (Figure S5). To determine if this enrichment for intergenic H3K56ac near initiation zones is associated with the promoters of expressed genes, we analyzed the distribution of expressed genes (regardless of epigenetic modifications) across replicons. This analysis showed that expressed genes are uniformly distributed (Figure S4), allowing us to conclude that much of the intergenic H3K56ac is associated with early replication and not gene expression.

## Discussion

### DNA replication and genome size

DNA replication has been profiled in *Drosophila*, mouse and human genomes [7]–[14], [22], [23], [60], [75]. *Arabidopsis* and *Drosophila* have a similar genome size (∼120 Mb each) and gene density (250 and 111 genes/Mb respectively) [54], [57], so it is not surprising that their replication profiles are similar. In contrast, the human and mouse genomes are substantially larger (3300 and 2500 Mb respectively) and have a much lower gene density (10 genes/Mb each) [55], [56]. Mammalian genomes are also characterized by large regions of uniform GC and gene content known as isochores [62], [76], [77]. In both human and mouse cells, replication time has been shown to correlate with isochore structure, and high GC, gene-rich isochores tend to replicate early in S phase [13], [62]. In contrast, it is not clear that a functionally equivalent isochore structure exists in *Arabidopsis* or *Drosophila* [77], [78]. Such differences in genome structure may explain why gene content and expression and the associated epigenetic modifications have a more subtle influence on replication time in *Arabidopsis* than in mammals. For example, in human cells, distance to the closest expressed gene is strongly correlated with replication time [23]. However, these distances are on the order of megabases. Such a correlation is meaningless in *Arabidopsis* where the median intergenic distance is less than one kilobase [54], [61]. Accordingly, we tailored our analysis to suit this compact genome, revealing many similarites and a few differences in the DNA replication programs of these model systems.

### DNA replication in *Arabidopsis* is biphasic

A common approach to determine DNA replication timing utilizes the direct hybridization of BrdU labeled early and late S phase DNA to genomic tiling arrays to construct a replication profile that indicates the enrichment of a given sequence in early relative to late S phase [7]–[9], [13], [14]. In this approach, DNA replication in mid S phase is inferred rather than directly evaluated. We measured *Arabidopsis* DNA replication in early, mid, and late S phase cells in separate microarray experiments producing three independent replication profiles. This strategy revealed that the replication profiles for early and mid S phase cells are very similar to each other and clearly distinct from the late S phase profile.

The majority of euchromatin in chr4 replicates in early and mid S phase, and the bulk of the heterochromatin replicates in late S phase (Figure 2). Temporal separation of DNA replication for euchromatin and heterochromatin was first observed at least five decades ago in both plants and animals [25] and is consistent with recent findings in *Drosophila*, mouse and human cells [21]. Fiber autoradiography experiments in *Arabidopsis* identified two temporal classes of replicons but did not distinguish euchromatin from heterochromatin [48].

Surprisingly, there is little difference between the early and mid S phase replication profiles (Figure 2), even though FACS profiles for the early and mid S phase cells are distinct (Figure 1 and Figure S2). When interpreting these results, it is important to remember that while FACS takes a DNA content measurement for each cell, the replication profiles are derived from a population of cells. If DNA replication followed a totally random program, a population of early S phase cells could produce a replication profile that encompasses the entire genome. At the other extreme is a strict temporal program in which the order of origin activation is highly consistent between cells in a population. With our experimental design, such a program would produce early S phase profiles showing replication of approximately 20% of the genome, while mid S phase profiles would be distinct from the early S phase profiles because they would encompass an additional 30% of the genome.

Our results are an intermediate case between these two extremes and are best explained by a biphasic model of replication for *Arabidopsis*. In this model, the bulk of euchromatin replicates in early to mid S phase and the heterochromatin replicates late. Origin utilization is largely the same in early and mid S phase, suggesting that the temporal order of origin activation in the first half of S phase is stochastic. While we did not attempt to identify origins *per se*, we did identify initiation zones, and we detected few, if any, initiation zones specific to mid S phase cells (Figure 3C and Figure 4B). The segmentation analysis showed some merging of early S phase segments to form larger mid S phase segments, but this effect most likely reflects elongation of replicons rather than activation of additional origins (Figure 3). The relative enrichment for initiation zones is similar in early and mid S phase cells suggesting that there is no quantitative difference in origin activation (Figure 3C). In contrast, there are many putative initiation zones specific for late S phase (Figure 3C and Figure 4B).

The idea that DNA replication follows a strict temporal program derives largely from seminal work in budding yeast [6], [79]. Budding yeast is characterized by sequence specific origins in a compact genome and, as such, might not be a good model for eukaryotes with much larger genomes and no clear origin sequence specificity [1]–[4]. Single molecule studies showed that even in budding and fission yeast, origin activation is stochastic and varies from cell to cell in a population [15], [16]. Whole genome studies in *Drosophila* and mouse cells are also consistent with a biphasic model of DNA replication. In *Drosophila*, initiation zones are most abundant in early and late S phase [14], while mouse replicons and replication domains tend to segment as either early or late [13]. Increasingly, origin activation is being interpreted as a largely stochastic process at the level of individual cells, with temporal profiles corresponding to the most probable sequence of origin activation for a population of cells [17], [18], [20].

### Replication time correlates with chromatin conformation

The replication time of any given DNA segment is related to three factors – distance from the closest origin, activation time of that origin, and rate of DNA elongation upstream of the segment. Chromatin conformation can influence the latter two factors, and chromatin remodeling factors have been shown to be critical for DNA replication [80]–[83]. Our analyses of replication time with respect to both genetic and epigenetic features revealed correlations that may reflect the effect of chromatin conformation on origin specification, origin activity and the rate of DNA elongation.

The heterochromatic knob and pericentromeric heterochromatin are entirely late replicating (Figure 4A). Both of these regions are depleted in genes, rich in TEs, and display abundant H3K9me2 and 5mC (Figure 2 and Table S11). This constitutive heterochromatin exists in a compact conformation throughout most of the cell cycle [59]. This conformation likely restricts both origin activation and DNA elongation [2]–[4]. In both budding and fission yeast, pericentromeric heterochromatin replicates in early S phase [6], [26], but pericentromeric DNA replicates in late S phase in animal cells [9], [84], [85]. In both cases, replication of heterochromatin is dependent on chromatin remodeling complexes [80], [82], [83], and it will be interesting to identify the complexes utilized by plants.

We focused our analyses on the long arm of chr4 because it represents a large contiguous, genomic segment generally regarded as euchromatic [33], [34], [58], [59]. However, we were surprised by the predominance of late replication in the proximal portion of the long arm (Figure 2 and Figure 4A). Probe and replicon level analyses revealed that relative to the distal long arm, the proximal long arm has considerable heterochromatic character, including decreased gene coverage/content, increased TE coverage/content, and elevated levels of both H3K9me2 and DNA 5mC (Figure 2, Table 1, and Table 2). Much of the proximal long arm likely adopts a chromatin state known as cryptic or facultative heterochromatin [28], [65]. Such regions share some of the biochemical features of constitutive heterochromatin, including hypoacetylation, H3K9me2 and DNA 5mC, but do not adopt the long range, highly condensed structure of constitutive heterochromatin. In mouse cells, replication domains that switch replication time upon differentiation are believed to be facultative heterochromatin [13], [21].

Despite the overall differences in replication time for the proximal long and distal long arm regions, we detected several correlations between replication time and genetic and epigenetic features that were similar in both regions. For example, EM-replicating probes show increased AT content, decreased gene coverage and decreased DNA 5mC (Table 1). Further, the histone modifications, H3K4me1/2 and H3K9me2, are decreased while H3K56ac is increased. The pattern is opposite for L-replicating probes. These observations suggest that DNA replication initiates in AT-rich intergenic regions with an open chromatin conformation and proceeds into regions where the epigenetic modifications associated with gene expression specify a more complex chromatin conformation. The distribution of genetic and epigenetic features within replicons further supports this hypothesis (Figure 5).

The EM replicons display trends that are consistent with a replicon model that has been termed the “relaxed replicon” model [2]–[4]. This model incorporates several mechanisms to explain ORC binding and replicon structure in higher eukaryotes. Mechanisms consistent with our work include a higher affinity of ORC for open chromatin and AT rich sequences [86], [87], transcriptional interference preventing ORC binding [88], and inhibition of ORC binding by DNA methylation [89]. The structure of EM replicons may be driven by the probability of both ORC binding and origin activation. Regions proximal to initiation zones have a higher AT content and elevated H3K56ac and may have a higher probability of binding ORC to form an origin (Figure 5). The lower gene density, lower H3K4me1/2 and reduced 5mC in these regions would also favor origin formation. Termination zones show opposite trends for these characteristics, consistent with a lower probability of binding ORC. In addition, elevated levels of H3K4me1/2 and 5mC may impede the progress of replication forks in these regions. Chromatin modified by DNA 5mC adopts a more compact conformation and impedes the progress of RNA polymerase [63], [90].

The trends for EM replicons are readily apparent when the epigenetic modifications are integrated over large regions (Figure 6). Most of the trends do not hold for L replicons, which in comparison to EM replicons, have greatly elevated and evenly distributed levels of H3K9me2 and 5mC indicative of a heterochromatic state (Table 2, Figure 5, and Figure 6). Replication may be delayed in these regions because it requires the activity of chromatin remodeling complexes, as discussed above for the heterochromatic knob and pericentromere. Additionally, L replicons may have a lower density of potential origins.

### Gene expression and replication time

Gene expression shows a positive correlation with early replication in all higher eukaryotes examined to date [7]–[9], [11]–[14], [22], [23]. This correlation is strongest when integrated over large regions because there are many exceptions at the level of individual genes. We identified a similar correlation in *Arabidopsis*, with genes in EM replicating regions more likely to be expressed than genes in L replicating regions (Table 4). However, the relationship of specific epigenetic modifications to gene expression and replication time is complex (Table 3). From the standpoint of replication time, two effects are prominent. H3K56ac with a lack of H3K9me2 is favorable for both gene expression and early replication, whereas H3K9me2 with a lack of H3K56ac correlates with lower expression and late replication. Genes associated with both H3K9me2 and H3K56ac also tend toward low expression and late replication, but the effect is less clear-cut than H3K9me2 alone. Genes with both marks are similar to the “pan S” or “biphasic” genes in human cells which bear both active and repressive chromatin marks due to interallelic variation [12], [60]. We also observed an increase in EML replication for these genes in *Arabidopsis* (Table 3). Unlike the epigenetic modifications discussed above, integration of gene expression over large regions did not reveal a correlation between gene expression and replicon structure (Figure 6). This lack of correlation probably reflects the fact that the expression of an individual gene is more strongly modulated by epigenetic modifications specific to that gene rather than by the global characteristics of large regions containing many genes.

### H3K56 acetylation and early replication

H3K56ac is thought to occur on all newly synthesized H3 histones and be required for nucleosome assembly [66], [69], [91]. H3K56ac is associated with regions of nucleosome exchange such as active promoters [70], [71], sites of DNA repair [67], [69], and nascent chromatin [40], [68]. In budding yeast, H3K56ac is most abundant during S phase and localizes to early origins in a cell cycle dependent manner [40], [68], [69]. Intriguingly, H3K56ac correlated with EM replication and was enriched at the center of *Arabidopsis* replicons (Figure 5 and Figure 6). Interpretation of this data must be tempered by the fact that the epigenetic profiling was performed on an unsorted population of cells so both replication dependent and independent H3K56ac is represented. Although there was a positive correlation between H3K56ac and gene expression (Table 3), integration of H3K56ac over large regions, including intergenic regions, showed a clear association with replication time and not with gene expression (Figure 6). H4K16ac correlates with early replication in *Drosophila* [14], while H3K56ac associates with early origins in budding yeast [40]. We have provided the first evidence, to our knowledge, linking H3K56ac to replication time in a higher eukaryote. Unlike H4K16, H3K56 is located in the core of the histone and is inaccessible to acetylation in the context of a fully assembled nucleosome [66], [92]. Therefore, H3K56ac might be associated with nascent DNA behind active replication forks rather than the disassembly of chromatin ahead of replication forks [68]. Nevertheless, H3K56ac may prove to be a valuable epigenetic mark for identifying replication origins.

### Conclusions

We have presented a high-resolution analysis of the replication program for a plant chromosome. *Arabidopsis* DNA replication is biphasic, with euchromatin replicating in the first half of S phase and heterochromatin replicating in the last half. This pattern is similar to other eukaryotes [9], [84], [85], although exceptions do occur in yeast [6], [26]. Within each phase, origin activation appears to be largely stochastic because we could discern few differences between replication profiles for early and mid S phase cells. This result provides additional support for the emerging model of stochastic origin activation rather than strict temporal regulation [15]–[18], [20]. The replication profiles allowed us to construct a replicon map for chr4 and to correlate replication time with gene expression and specific epigenetic modifications. We showed that initiation zones are enriched for epigenetic features associated with open chromatin, providing support for the “relaxed replicon” model, which proposes that origin specification and activity are strongly influenced by both sequence content and chromatin conformation in higher eukaryotes [1]–[4]. Finally, we showed that early replicating regions and initiation zones are enriched for H3K56ac. This histone modification continues to be an area of intense research because of its role in DNA replication, DNA repair and gene expression. We provide evidence that H3K56ac has both replication independent and dependent roles in plants by showing that genes bearing H3K56ac have a higher probability of expression, whereas large regions with elevated H3K56ac levels are associated with early replication. Replication time and H3K56ac data in conjunction with other experiments may help us identify replication origins in plants. This study linking DNA replication and replicon structure to chromatin conformation provides a foundation for future studies that will investigate the impact of these processes on plant growth and development.

## Materials and Methods

### Cell culture

The *Arabidopsis* cell line (Col-0, ecotype Columbia) was maintained in Gamborg's B5 basal medium with minor salt (Sigma G5893) supplemented with 1.1 mg/L 2,4-dichlorophenoxyacetic acid, 3 mM MES and 3% sucrose. The cells were grown on a rotary shaker at 160 rpm under constant light at 23°C and subcultured every 7 days with a 1∶10 (inoculum∶fresh medium) dilution [34].

BrdU labeling was maximized using a ‘7-d split culture' by mixing 25 mL of fresh medium and 25 mL of the *Arabidopsis* culture at 7 days post subculture. The 7-d split culture was grown for 16 h and then labeled for 1 h with 100 µM BrdU (Sigma B9285). Labeled cells were fixed in 1% paraformaldehyde for 15 min, washed in 1× phosphate buffered saline (PBS) three times, and snap frozen in liquid nitrogen. Time course experiments showed that BrdU incorporation is highest between 12 and 16 h post-labeling (Figure S1C). Cells from six 7-d split cultures were combined for each biological replicate.

### Nuclei isolation

The frozen cell pellet for each biological replicate was ground at 4°C in 150 mL lysis buffer (15 mM Tris-HCl pH 7.5, 2 mM EDTA, 80 mM KCl, 20 mM NaCl, 15 mM β-mercaptoethanol, and 0.1% Triton X-100) using a commercial blender. The ground cell suspension was incubated at 4°C for 5 min and filtered through a 3-tiered nylon mesh (100, 50, and 30 µm). The filtrate was centrifuged at 200 ×g for 5 min at 4°C, and the nuclei were resuspended in 8 mL of lysis buffer containing 2 µg/mL DAPI and 50 µg/mL RNase A. The isolated nuclei were filtered through a 20-µm nylon filter before flow cytometric analysis and sorting.

### FACS analysis and flow sorting

Nuclei were sorted and recovered using an InFlux cell sorter (BD Biosciences) equipped with a 355-nm UV laser and a 488-nm sapphire laser. STE buffer (10 mM Tris-HCl pH 7.5, 1 mM EDTA, and 100 mM NaCl) was used as a sheath fluid, and nuclei were sorted into a 50-mL tube containing 5 mL STE buffer.

An analytical FACS profile for BrdU incorporation and DNA content was generated as described [93] with some modifications. BrdU-labeled cells were fixed in 70% ethanol on ice for 1 h and frozen in liquid nitrogen. Nuclei were isolated, denatured in 2N HCl and 0.5% Triton X-100 at room temperature for 30 min, neutralized by adding 0.1 M Na_2_B_4_O_7_ (pH 8.5), and washed twice with PBS-TBR (1x PBS, 1% BSA, 0.5% Tween-20 and 50 µg/mL RNase A). The nuclei were resuspended in PBS-TBR containing a 1∶50 dilution of anti-BrdU Alexa Fluor 488 conjugate (Invitrogen) by gentle agitation overnight at 4°C in the dark. The nuclei were washed once with PBS-TBR, incubated in PBS-TBR containing 10 µg/mL propidium iodide for at least 30 min, filtered through a 20-µm nylon filter, and analyzed by FACS. FlowJo (Version 8.8.6) software was used for the data analysis.

### Genomic DNA extraction from sorted nuclei

To reverse the crosslinks, the sorted nuclei were treated with 50 mM EDTA, 1% sodium lauroyl sarcosine and 200 µg/mL proteinase K for 1 h at 42°C and then overnight at 65°C in the dark. The mixture was supplemented with 4 mg/mL phenylmethanesulphonylfluoride and incubated for 40 min at room temperature prior to extraction of genomic DNA using phenol/chloroform/IAA in a phase lock gel (Sigma). The upper aqueous phase was mixed with 150 µg/mL GlycoBlue (Ambion) and precipitated with 0.3 M sodium acetate and 2 volumes of cold ethanol. The DNA was centrifuged and the pellet was washed with 70% ethanol once, dried for 5 min using a SpeedVac in the dark, and resuspended in sterile water.

### BrdU immunoprecipitation

BrdU-labeled DNA was immunoprecitated as described [94] with some minor modifications. Genomic DNA extracted from the sorted nuclei was sonicated in 450 µL of ChIP dilution buffer (0.1% BSA, 1.2 mM EDTA, 16.7 mM Tris-HCl pH 8, and 167 mM NaCl) to a shear-size of 500 to 1000 bp, followed by addition of Triton X-100 (1.1%). The sheared DNA was denatured at 95°C for 5 min and immediately cooled on ice for at least 5 min. One mL of cold ChIP dilution buffer containing 1.1% Triton X-100 was added and the sheared DNA was incubated with 0.5 µL anti-BrdU antibody (Invitrogen) for 3 h at 4°C. DNA containing BrdU was immunoprecipitated by adding 100 µL of 50% protein G-sepharose beads (Sigma) and incubating overnight in the dark at 4°C with gentle agitation. The beads were washed as previously described by Gendrel, et al. (2005). BrdU-labeled DNA was eluted from the beads with 0.2 M glycine (pH 2.5) and neutralized by adding 10% (v/v) of 1 M Tris-HCl (pH 8). Eluted DNA was treated with proteinase K for 1 h at 45°C, extracted with phenol/chloroform/IAA, and precipitated with sodium acetate and ethanol. Precipitated DNA was resuspended in RT-PCR grade water (Ambion) and used as template for random amplification and real-time quantitative PCR.

### Microarray hybridization

BrdU immunoprecipitated DNA (target DNA) and input DNA (reference DNA) samples were amplified as described [95], purified and concentrated to 200–250 ng/µL using a QIAquick PCR Purification Kit (QIAGEN). Each amplified DNA sample (1.5 mg) was labeled with either Cy3 or Cy5 fluorescent dye and purified using a BioPrime Array CGH Genomic Labeling System (Invitrogen). The Cy dye-labeled target and reference samples were co-hybridized on a custom-printed tiling array [33] with a dye-swap experimental design. Each experiment comprised six microarrays representing the three biological replicates and the corresponding dye swaps. Microarray hybridization and washing were previously described [34] but modified to include DyeSaver2 coating reagent (Genisphere) to minimize oxidation of Cy5. Hybridized microarrays were scanned using a PerkinElmer ScanArray Express scanner and quantified using GenePix Pro software (version 6.01).

### Microarray data normalization and analysis of replication time

Calculation of microarray probe enrichment ratios, loess and quantile normalizations were done in the R statistical computing environment with the limma package using default settings [96]–[98]. Probe ratios were loess-smoothed in a 150-kb window for replication profiles and identification of initiation and termination zones. Segments of contiguous replication time were defined as regions where smoothed probe ratios were greater than zero for a minimum of 10-kb. This filter minimized excessive replication time changes in regions with low probe enrichment ratios. Merging of the segmentations for early, mid and late S phase cells was done by determining the regions of overlap. The 10-kb length minimum was not used at this step. Initiation and termination zones were identified as the inflection points of the loess-smoothed profiles as described in the results. Zones were then defined as the 10-kb regions centered at the inflection point. Overlapping zones were merged into a single zone. Replication boundaries were chosen from the three sets of termination zones based on the following order of precedence: 1) termination zones present in early, mid and late S phase cells, 2) termination zones enriched in late S phase and 3) termination zones that manifest as local minima but enriched in early and/or mid S phase.

### Bioinformatics and statistical analysis

All data manipulation and statistical analysis was performed with R and Bioconductor [96], [99]. A database incorporating probe ratios for replication time, histone modifications, DNA 5mC and the TAIR8 *Arabidopsis* genome annotation [61] was constructed to facilitate analysis. Gene and TE coverage values for probes and larger regions are the percentage of bases in that region that overlap with any gene or TE respectively. Overlapping genes or TEs were treated as one so that coverage values do not exceed 100%. Statistical comparisons of GC content and gene or TE coverage were performed by one-sample t-tests. AT-rich and gene-rich probes were defined as the top quartile of all probes on the array. AT-rich, gene-rich and probes positive for histone modifications or DNA methylation data were treated as binomial data, and a one-sample binomial test was used for analyses. Gene expression values were determined using the affy package in R [73]. MAS5 presence or absence calls and gcRMA expression values were calculated using default settings. The pattern of epigenetic modifications for chr4 genes was determined from the modifications of the overlapping probes again treating the modifications as binomial data. Heat maps for epigenetic modifications were generated by smoothing probe ratios in a 150-kb window as for replication profiles and ranking the data by deciles for the whole of chr4. Heat maps for gene expression were generated similarly but gcRMA expression values were used rather than probe ratios. R scripts for all analyses and figures are available upon request.

## Supporting Information

Figure S1 Optimization of the *Arabidopsis* cell suspension culture conditions. (A) Standard growth curve of the cell suspension culture. Shown are the mean ± SE from two biological replicates. The suspension culture is subcultured weekly with 1∶10 dilution. (B) Typical cell morphology of cells in a 7-d split culture. Horizontal bar is 20 µm in length. (C) BrdU incorporation of cells taken at 6 different time points after 1∶1 ratio subculture of 7-d old culture (7-d split). The BrdU quantitation of pulse-labeled genomic DNA was performed by BrdU dot blot assay as described in Text S1. Shown are the mean ± SE from three biological replicates. (D) Nuclei isolated from a 7-d split culture at 16 hrs. The nuclei were used for flow sorting to profile early, mid, and late replication. For microscopy, the nuclei were diluted 20-fold. Horizontal bar is 10 µm in length.
(2.61 MB PDF)

Figure S2 FACS reanalysis of sorted nuclei from early/G1, mid S and late S/G2/M cells. (A) Composite FACS reanalysis of nuclei from previously sorted populations representing early S/G1, mid S, and late S/G2/M. (B) A pseudo-color representation of the BrdU incorporation and DNA content of nuclei from the mid S population of Figure 1A in the main text is compared with a histogram of the DNA content distribution in the early S/G1 sample in (A) (blue line). Mid S phase nuclei in the early S/G1 sort have a DNA content from the lower tail of the mid S phase population (shaded pink).
(0.11 MB PDF)

Figure S3 Real time qPCR validation of replication time microarray data. Five primer sets for early and late replicating regions (A,B, respectively) and six for mid replicating regions (C) were used to validate the microarray results (See Table S3 for positions). The barplots show the mean fold change with error bars for the qPCR data indicating ± SE for the three biological replicates. Each qPCR reaction was repeated twice with unamplified IP DNA from each biological replicate.
(0.09 MB PDF)

Figure S4 Distribution of genes with select epigenetic patterns within replicons for the long arm of chr4. The epigenetic pattern of chr4 genes was determined from the overlapping probes. Genes with pattern 3 show a slight enrichment near the initiation zones of EM replicons. Genes with patterns 1, 2 and 4 are uniformly distributed across replicons as are all expressed gene, regardless of epigenetic pattern.
(0.12 MB PDF)

Figure S5 Distribution of intergenic regions with select epigenetic patterns within replicons for the long arm of chr4. Intergenic regions were defined as those regions that did not overlap with any annotated gene. The epigenetic pattern of these regions was determined from the overlapping probes. The two most abundant epigenetic patterns for intergenic regions were patterns 3 and 13. Pattern 3 shows a clear asymmetric distribution across EM replicons whereas regions with pattern 13 are evenly distributed.
(0.11 MB PDF)

Table S1 Analysis of S phase nuclei in sorted populations of *Arabidopsis* suspension cells.
(0.04 MB DOC)

Table S2 Reanalysis of sorted populations of nuclei for estimation of purity.
(0.06 MB DOC)

Table S3 Sequence and chromosome position of real time qPCR primer sets used for microarray data validation.
(0.07 MB DOC)

Table S4 Real time qPCR validation of the enriched and depleted regions at different replication timings identified by microarray analysis.
(0.06 MB DOC)

Table S5 Segments of coordinate replication time for chr4.
(0.03 MB XLS)

Table S6 Distribution of probe class with respect to replication time.
(0.03 MB XLS)

Table S7 Analysis of initiation and termination zones between early, mid and late S phase cells.
(0.02 MB XLS)

Table S8 Coordinates and replication time of initiation zones identified in early, mid, and late S phase cells.
(0.06 MB XLS)

Table S9 Coordinates, initiation zones and replication time for chr4 replicons.
(0.06 MB XLS)

Table S10 Coordinates and replication time for chr4 replication domains.
(0.02 MB XLS)

Table S11 Probe-level analysis of replication time and epigenetic and genetic features for all chr4 regions.
(0.03 MB XLS)

Table S12 Epigenetic patterns for chr4 intergenic regions.
(0.02 MB XLS)

Text S1 BrdU and qPCR materials and methods.
(0.05 MB DOC)

## References

[1] Berezney R, Dubey DD, Huberman JA (2000). Heterogeneity of eukaryotic replicons, replicon clusters, and replication foci.. Chromosoma.

[2] Gilbert DM (2004). In search of the holy replicator.. Nat Rev Mol Cell Biol.

[3] Aladjem MI (2007). Replication in context: dynamic regulation of DNA replication patterns in metazoans.. Nat Rev Genet.

[4] Hamlin JL, Mesner LD, Lar O, Torres R, Chodaparambil SV (2008). A revisionist replicon model for higher eukaryotic genomes.. J Cell Biochem.

[5] Gondor A, Ohlsson R (2009). Replication timing and epigenetic reprogramming of gene expression: a two-way relationship?. Nat Rev Genet.

[6] Raghuraman MK, Winzeler EA, Collingwood D, Hunt S, Wodicka L (2001). Replication dynamics of the yeast genome.. Science.

[7] MacAlpine DM, Rodriguez HK, Bell SP (2004). Coordination of replication and transcription along a *Drosophila* chromosome.. Genes Dev.

[8] Schubeler D, Scalzo D, Kooperberg C, van Steensel B, Delrow J (2002). Genome-wide DNA replication profile for *Drosophila melanogaster*: a link between transcription and replication timing.. Nat Genet.

[9] White EJ, Emanuelsson O, Scalzo D, Royce T, Kosak S (2004). DNA replication-timing analysis of human chromosome 22 at high resolution and different developmental states.. Proc Natl Acad Sci U S A.

[10] Woodfine K, Fiegler H, Beare DM, Collins JE, McCann OT (2004). Replication timing of the human genome.. Hum Mol Genet.

[11] Jeon Y, Bekiranov S, Karnani N, Kapranov P, Ghosh S (2005). Temporal profile of replication of human chromosomes.. Proc Natl Acad Sci U S A.

[12] Karnani N, Taylor C, Malhotra A, Dutta A (2007). Pan-S replication patterns and chromosomal domains defined by genome-tiling arrays of ENCODE genomic areas.. Genome Res.

[13] Hiratani I, Ryba T, Itoh M, Yokochi T, Schwaiger M (2008). Global reorganization of replication domains during embryonic stem cell differentiation.. PLoS Biol.

[14] Schwaiger M, Stadler MB, Bell O, Kohler H, Oakeley EJ (2009). Chromatin state marks cell-type- and gender-specific replication of the *Drosophila* genome.. Genes Dev.

[15] Czajkowsky DM, Liu J, Hamlin JL, Shao Z (2008). DNA combing reveals intrinsic temporal disorder in the replication of yeast chromosome VI.. J Mol Biol.

[16] Patel PK, Arcangioli B, Baker SP, Bensimon A, Rhind N (2006). DNA replication origins fire stochastically in fission yeast.. Mol Biol Cell.

[17] Rhind N (2006). DNA replication timing: random thoughts about origin firing.. Nat Cell Biol.

[18] Lygeros J, Koutroumpas K, Dimopoulos S, Legouras I, Kouretas P (2008). Stochastic hybrid modeling of DNA replication across a complete genome.. Proc Natl Acad Sci U S A.

[19] Goldar A, Labit H, Marheineke K, Hyrien O (2008). A dynamic stochastic model for DNA replication initiation in early embryos.. PLoS ONE.

[20] Goldar A, Marsolier-Kergoat MC, Hyrien O (2009). Universal temporal profile of replication origin activation in eukaryotes.. PLoS ONE.

[21] Hiratani I, Takebayashi S, Lu J, Gilbert DM (2009). Replication timing and transcriptional control: beyond cause and effect—part II.. Curr Opin Genet Dev.

[22] Farkash-Amar S, Lipson D, Polten A, Goren A, Helmstetter C (2008). Global organization of replication time zones of the mouse genome.. Genome Res.

[23] Desprat R, Thierry-Mieg D, Lailler N, Lajugie J, Schildkraut C (2009). Predictable dynamic program of timing of DNA replication in human cells.. Genome Res.

[24] Donaldson AD (2005). Shaping time: chromatin structure and the DNA replication programme.. Trends Genet.

[25] Lima-de-Faria A, Jaworska H (1968). Late DNA synthesis in heterochromatin.. Nature.

[26] Kim SM, Dubey DD, Huberman JA (2003). Early-replicating heterochromatin.. Genes Dev.

[27] Cedar H, Bergman Y (2009). Linking DNA methylation and histone modification: patterns and paradigms.. Nat Rev Genet.

[28] Fransz P, ten Hoopen R, Tessadori F (2006). Composition and formation of heterochromatin in *Arabidopsis thaliana*.. Chromosome Res.

[29] Henderson IR, Jacobsen SE (2007). Epigenetic inheritance in plants.. Nature.

[30] Barski A, Cuddapah S, Cui K, Roh TY, Schones DE (2007). High-resolution profiling of histone methylations in the human genome.. Cell.

[31] Lippman Z, Gendrel AV, Black M, Vaughn MW, Dedhia N (2004). Role of transposable elements in heterochromatin and epigenetic control.. Nature.

[32] Mikkelsen TS, Ku M, Jaffe DB, Issac B, Lieberman E (2007). Genome-wide maps of chromatin state in pluripotent and lineage-committed cells.. Nature.

[33] Vaughn MW, Tanurdzic M, Lippman Z, Jiang H, Carrasquillo R (2007). Epigenetic natural variation in *Arabidopsis thaliana*.. PLoS Biol.

[34] Tanurdzic M, Vaughn MW, Jiang H, Lee TJ, Slotkin RK (2008). Epigenomic consequences of immortalized plant cell suspension culture.. PLoS Biol.

[35] Birney E, Stamatoyannopoulos JA, Dutta A, Guigo R, Gingeras TR (2007). Identification and analysis of functional elements in 1% of the human genome by the ENCODE pilot project.. Nature.

[36] Schubeler D, MacAlpine DM, Scalzo D, Wirbelauer C, Kooperberg C (2004). The histone modification pattern of active genes revealed through genome-wide chromatin analysis of a higher eukaryote.. Genes Dev.

[37] Zhang X, Bernatavichute YV, Cokus S, Pellegrini M, Jacobsen SE (2009). Genome-wide analysis of mono-, di- and trimethylation of histone H3 lysine 4 in *Arabidopsis thaliana*.. Genome Biol.

[38] Zhang X, Yazaki J, Sundaresan A, Cokus S, Chan SW (2006). Genome-wide high-resolution mapping and functional analysis of DNA methylation in *Arabidopsis*.. Cell.

[39] Goren A, Tabib A, Hecht M, Cedar H (2008). DNA replication timing of the human beta-globin domain is controlled by histone modification at the origin.. Genes Dev.

[40] Kaplan T, Liu CL, Erkmann JA, Holik J, Grunstein M (2008). Cell cycle- and chaperone-mediated regulation of H3K56ac incorporation in yeast.. PLoS Genet.

[41] Knott SR, Viggiani CJ, Tavare S, Aparicio OM (2009). Genome-wide replication profiles indicate an expansive role for Rpd3L in regulating replication initiation timing or efficiency, and reveal genomic loci of Rpd3 function in *Saccharomyces cerevisiae*.. Genes Dev.

[42] Jorgensen HF, Azuara V, Amoils S, Spivakov M, Terry A (2007). The impact of chromatin modifiers on the timing of locus replication in mouse embryonic stem cells.. Genome Biol.

[43] Lande-Diner L, Zhang J, Cedar H (2009). Shifts in replication timing actively affect histone acetylation during nucleosome reassembly.. Mol Cell.

[44] Falbo KB, Shen X (2009). Histone modifications during DNA replication.. Mol Cells.

[45] Vant' Hof J, DePamphilis ML (1996). DNA replication in plants.. DNA replication in eukaryotic cells.

[46] Shultz RW, Lee TJ, Allen GC, Thompson WF, Hanley-Bowdoin L (2009). Dynamic localization of the DNA replication proteins MCM5 and MCM7 in plants.. Plant Physiol.

[47] Shultz RW, Tatineni VM, Hanley-Bowdoin L, Thompson WF (2007). Genome-wide analysis of the core DNA replication machinery in the higher plants *Arabidopsis* and rice.. Plant Physiol.

[48] Vant' Hof J, Kuniyuki A, Bjerknes CA (1978). Size and number of replicon families of chromosomal DNA of *Arabidopsis thaliana*.. Chromosoma.

[49] Martienssen RA, Kloc A, Slotkin RK, Tanurdzic M (2008). Epigenetic inheritance and reprogramming in plants and fission yeast.. Cold Spring Harb Symp Quant Biol.

[50] Finnegan EJ, Dennis ES (2007). Vernalization-induced trimethylation of histone H3 lysine 27 at FLC is not maintained in mitotically quiescent cells.. Curr Biol.

[51] Finnegan EJ, Kovac KA (2000). Plant DNA methyltransferases.. Plant Mol Biol.

[52] Pandey R, Muller A, Napoli CA, Selinger DA, Pikaard CS (2002). Analysis of histone acetyltransferase and histone deacetylase families of *Arabidopsis thaliana* suggests functional diversification of chromatin modification among multicellular eukaryotes.. Nucleic Acids Res.

[53] Springer NM, Napoli CA, Selinger DA, Pandey R, Cone KC (2003). Comparative analysis of SET domain proteins in maize and *Arabidopsis* reveals multiple duplications preceding the divergence of monocots and dicots.. Plant Physiol.

[54] The *Arabidopsis* Genome Initiative (2000). Analysis of the genome sequence of the flowering plant *Arabidopsis thaliana*.. Nature.

[55] Lander ES, Linton LM, Birren B, Nusbaum C, Zody MC (2001). Initial sequencing and analysis of the human genome.. Nature.

[56] Waterston RH, Lindblad-Toh K, Birney E, Rogers J, Abril JF (2002). Initial sequencing and comparative analysis of the mouse genome.. Nature.

[57] Adams MD, Celniker SE, Holt RA, Evans CA, Gocayne JD (2000). The genome sequence of *Drosophila melanogaster*.. Science.

[58] Mayer K, Schuller C, Wambutt R, Murphy G, Volckaert G (1999). Sequence and analysis of chromosome 4 of the plant *Arabidopsis thaliana*.. Nature.

[59] Fransz P, De Jong JH, Lysak M, Castiglione MR, Schubert I (2002). Interphase chromosomes in *Arabidopsis* are organized as well defined chromocenters from which euchromatin loops emanate.. Proc Natl Acad Sci U S A.

[60] Hansen RS, Thomas S, Sandstrom R, Canfield TK, Thurman RE (2009). Sequencing newly replicated DNA reveals widespread plasticity in human replication timing.. Proc Natl Acad Sci U S A.

[61] Swarbreck D, Wilks C, Lamesch P, Berardini TZ, Garcia-Hernandez M (2008). The *Arabidopsis* Information Resource (TAIR): gene structure and function annotation.. Nucleic Acids Res.

[62] Costantini M, Bernardi G (2008). Replication timing, chromosomal bands, and isochores.. Proc Natl Acad Sci U S A.

[63] Zilberman D, Gehring M, Tran RK, Ballinger T, Henikoff S (2007). Genome-wide analysis of *Arabidopsis thaliana* DNA methylation uncovers an interdependence between methylation and transcription.. Nat Genet.

[64] Gendrel AV, Lippman Z, Yordan C, Colot V, Martienssen RA (2002). Dependence of heterochromatic histone H3 methylation patterns on the *Arabidopsis* gene DDM1.. Science.

[65] Trojer P, Reinberg D (2007). Facultative heterochromatin: is there a distinctive molecular signature?. Mol Cell.

[66] Xu F, Zhang K, Grunstein M (2005). Acetylation in histone H3 globular domain regulates gene expression in yeast.. Cell.

[67] Das C, Lucia MS, Hansen KC, Tyler JK (2009). CBP/p300-mediated acetylation of histone H3 on lysine 56.. Nature.

[68] Li Q, Zhou H, Wurtele H, Davies B, Horazdovsky B (2008). Acetylation of histone H3 lysine 56 regulates replication-coupled nucleosome assembly.. Cell.

[69] Masumoto H, Hawke D, Kobayashi R, Verreault A (2005). A role for cell-cycle-regulated histone H3 lysine 56 acetylation in the DNA damage response.. Nature.

[70] Rufiange A, Jacques PE, Bhat W, Robert F, Nourani A (2007). Genome-wide replication-independent histone H3 exchange occurs predominantly at promoters and implicates H3 K56 acetylation and Asf1.. Mol Cell.

[71] Williams SK, Truong D, Tyler JK (2008). Acetylation in the globular core of histone H3 on lysine-56 promotes chromatin disassembly during transcriptional activation.. Proc Natl Acad Sci U S A.

[72] Hubbell E, Liu WM, Mei R (2002). Robust estimators for expression analysis.. Bioinformatics.

[73] Gautier L, Cope L, Bolstad BM, Irizarry RA (2004). affy—analysis of Affymetrix GeneChip data at the probe level.. Bioinformatics.

[74] Xie W, Song C, Young NL, Sperling AS, Xu F (2009). Histone h3 lysine 56 acetylation is linked to the core transcriptional network in human embryonic stem cells.. Mol Cell.

[75] Woodfine K, Beare DM, Ichimura K, Debernardi S, Mungall AJ (2005). Replication timing of human chromosome 6.. Cell Cycle.

[76] Bernardi G (2000). Isochores and the evolutionary genomics of vertebrates.. Gene.

[77] Oliver JL, Bernaola-Galvan P, Carpena P, Roman-Roldan R (2001). Isochore chromosome maps of eukaryotic genomes.. Gene.

[78] Zhang R, Zhang CT (2004). Isochore structures in the genome of the plant Arabidopsis thaliana.. J Mol Evol.

[79] Friedman KL, Brewer BJ, Fangman WL (1997). Replication profile of *Saccharomyces cerevisiae* chromosome VI.. Genes Cells.

[80] Collins N, Poot RA, Kukimoto I, Garcia-Jimenez C, Dellaire G (2002). An ACF1-ISWI chromatin-remodeling complex is required for DNA replication through heterochromatin.. Nat Genet.

[81] Falbo KB, Shen X (2006). Chromatin remodeling in DNA replication.. J Cell Biochem.

[82] Quivy JP, Gerard A, Cook AJ, Roche D, Almouzni G (2008). The HP1-p150/CAF-1 interaction is required for pericentric heterochromatin replication and S-phase progression in mouse cells.. Nat Struct Mol Biol.

[83] Vincent JA, Kwong TJ, Tsukiyama T (2008). ATP-dependent chromatin remodeling shapes the DNA replication landscape.. Nat Struct Mol Biol.

[84] Takebayashi S, Sugimura K, Saito T, Sato C, Fukushima Y (2005). Regulation of replication at the R/G chromosomal band boundary and pericentromeric heterochromatin of mammalian cells.. Exp Cell Res.

[85] Wu R, Singh PB, Gilbert DM (2006). Uncoupling global and fine-tuning replication timing determinants for mouse pericentric heterochromatin.. J Cell Biol.

[86] Vashee S, Cvetic C, Lu W, Simancek P, Kelly TJ (2003). Sequence-independent DNA binding and replication initiation by the human origin recognition complex.. Genes Dev.

[87] Stanojcic S, Lemaitre JM, Brodolin K, Danis E, Mechali M (2008). In *Xenopus* egg extracts, DNA replication initiates preferentially at or near asymmetric AT sequences.. Mol Cell Biol.

[88] Saha S, Shan Y, Mesner LD, Hamlin JL (2004). The promoter of the Chinese hamster ovary dihydrofolate reductase gene regulates the activity of the local origin and helps define its boundaries.. Genes Dev.

[89] Harvey KJ, Newport J (2003). CpG methylation of DNA restricts prereplication complex assembly in *Xenopus* egg extracts.. Mol Cell Biol.

[90] Lorincz MC, Dickerson DR, Schmitt M, Groudine M (2004). Intragenic DNA methylation alters chromatin structure and elongation efficiency in mammalian cells.. Nat Struct Mol Biol.

[91] Han J, Zhou H, Horazdovsky B, Zhang K, Xu RM (2007). Rtt109 acetylates histone H3 lysine 56 and functions in DNA replication.. Science.

[92] Han J, Zhou H, Li Z, Xu RM, Zhang Z (2007). The Rtt109-Vps75 histone acetyltransferase complex acetylates non-nucleosomal histone H3.. J Biol Chem.

[93] Dolbeare F, Gratzner H, Pallavicini MG, Gray JW (1983). Flow Cytometric Measurement of Total DNA Content and Incorporated Bromodeoxyuridine.. Proc Natl Acad Sci U S A.

[94] Gendrel AV, Lippman Z, Martienssen R, Colot V (2005). Profiling histone modification patterns in plants using genomic tiling microarrays.. Nature Methods.

[95] Lippman Z, Gendrel AV, Colot V, Martienssen R (2005). Profiling DNA methylation patterns using genomic tiling microarrays.. Nat Methods.

[96] Team RDC (2009). R: A language and environment for statistical computing..

[97] Smyth GK, Speed T (2003). Normalization of cDNA microarray data.. Methods.

[98] Smyth GK (2005). Limma: linear models for microarray data. In: Gentleman R, Carey V, Dudoit S, Irizarry R, Huber W, editors. Bioinformatics and Computational Biology Solutions using R and Bioconductor..

[99] Gentleman RC, Carey VJ, Bates DM, Bolstad B, Dettling M (2004). Bioconductor: open software development for computational biology and bioinformatics.. Genome Biol.

