# Supplementary figures and images for: Arabidopsis thaliana Chromosome 4 Replicates in Two Phases That Correlate with Chromatin State
Source: PLoS Genet. 2010 Jun 10;6(6):e1000982. doi: 10.1371/journal.pgen.1000982 (PMC2883604; doi:10.1371/journal.pgen.1000982)

Figure S1  
Lee and Pasccuzi

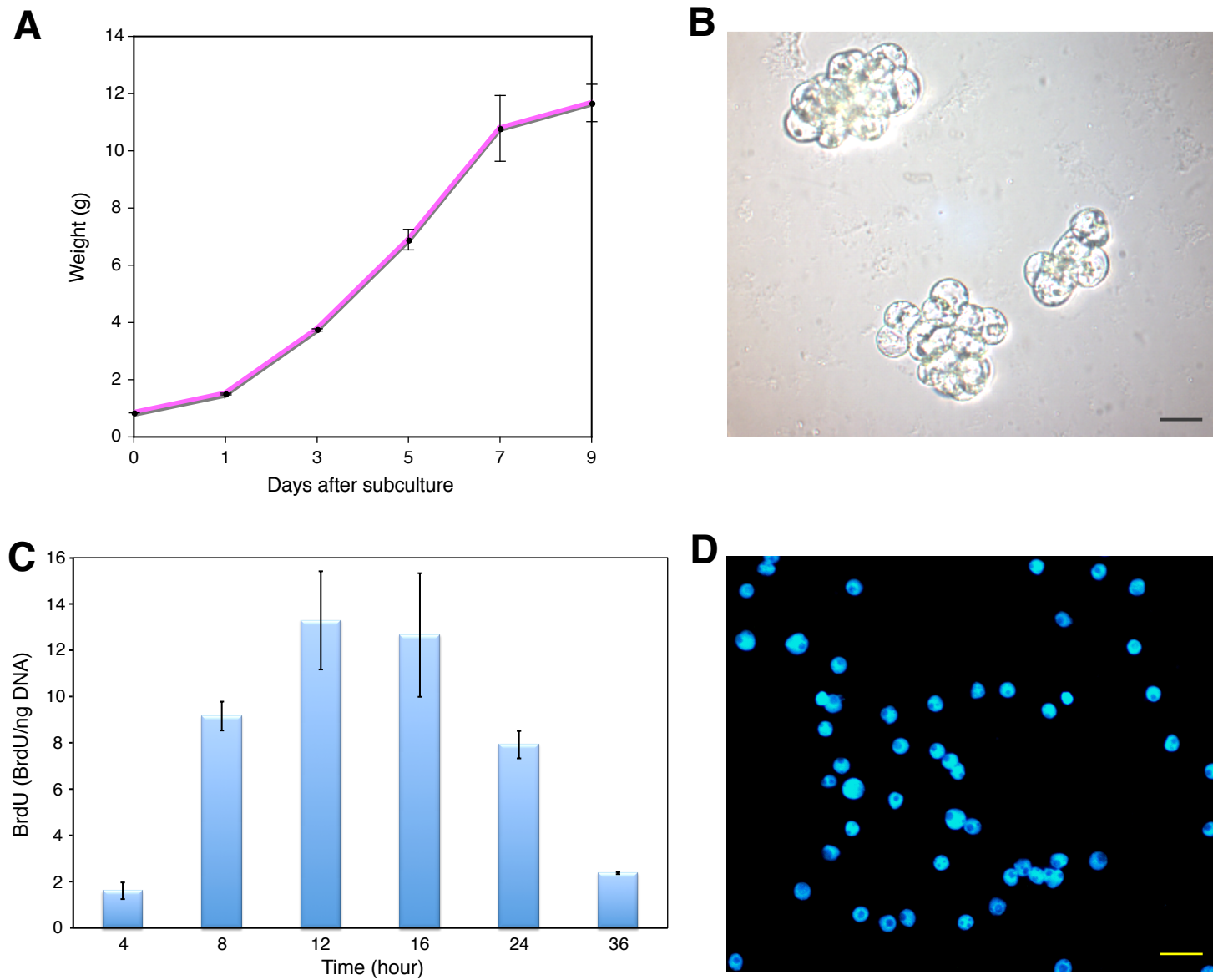

Supplement: Figure S1 — Optimization of the Arabidopsis cell suspension culture conditions. (A) Standard growth curve of the cell suspension culture. Shown are the mean ± SE from two biological replicates. The suspension culture is subcultured weekly with 1∶10 dilution. (B) Typical cell morphology of cells in a 7-d split culture. Horizontal bar is 20 µm in length. (C) BrdU incorporation of cells taken at 6 different time points after 1∶1 ratio subculture of 7-d old culture (7-d split). The BrdU quantitation of pulse-labeled genomic DNA was performed by BrdU dot blot assay as described in Text S1. Shown are the mean ± SE from three biological replicates. (D) Nuclei isolated from a 7-d split culture at 16 hrs. The nuclei were used for flow sorting to profile early, mid, and late replication. For microscopy, the nuclei were diluted 20-fold. Horizontal bar is 10 µm in length. (2.61 MB PDF) [file pgen.1000982.s001.pdf]

**Figure S2.** FACS reanalysis of sorted nuclei from early S/G1, mid S and late S/G2/M cells

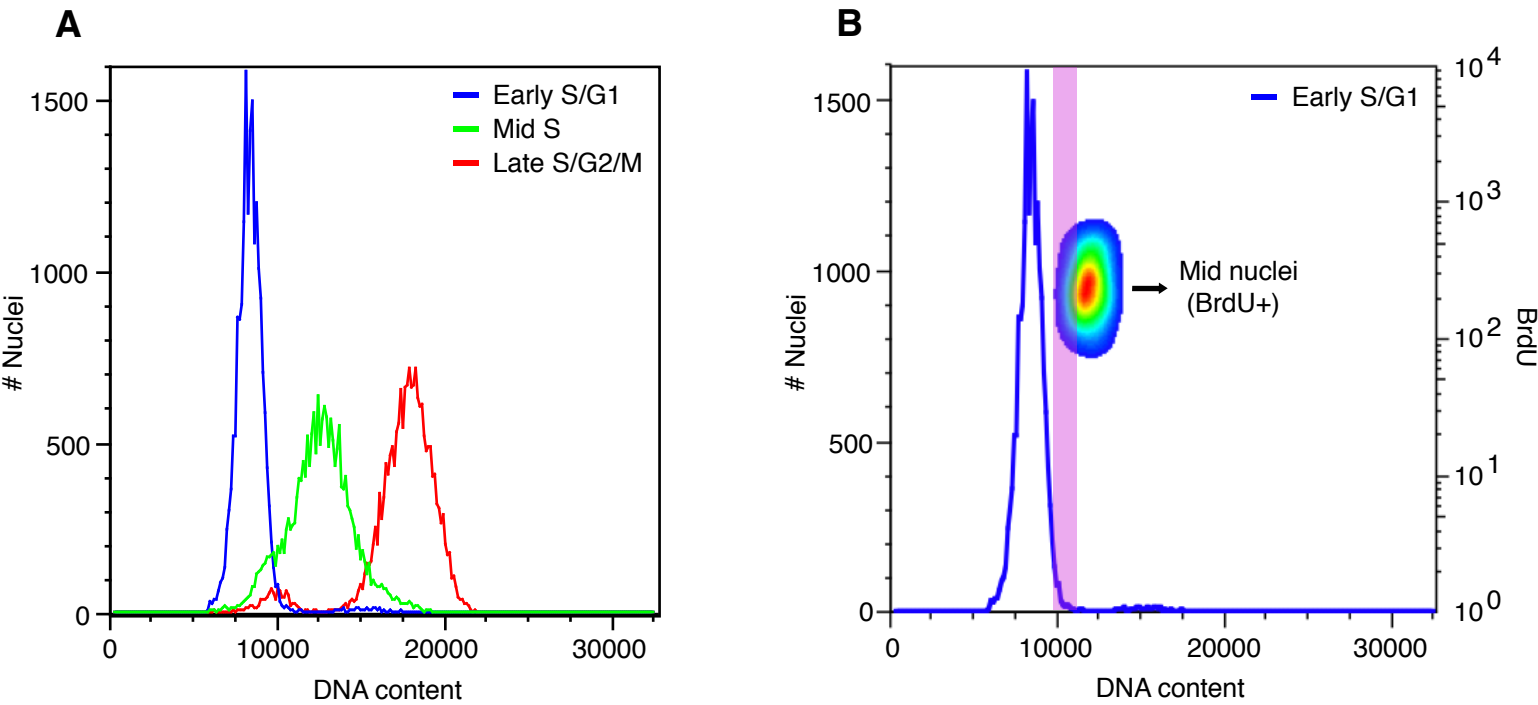

Supplement: Figure S2 — FACS reanalysis of sorted nuclei from early/G1, mid S and late S/G2/M cells. (A) Composite FACS reanalysis of nuclei from previously sorted populations representing early S/G1, mid S, and late S/G2/M. (B) A pseudo-color representation of the BrdU incorporation and DNA content of nuclei from the mid S population of Figure 1A in the main text is compared with a histogram of the DNA content distribution in the early S/G1 sample in (A) (blue line). Mid S phase nuclei in the early S/G1 sort have a DNA content from the lower tail of the mid S phase population (shaded pink). (0.11 MB PDF) [file pgen.1000982.s002.pdf]

**Figure S3.** Real time qPCR validation of replication timing microarray data

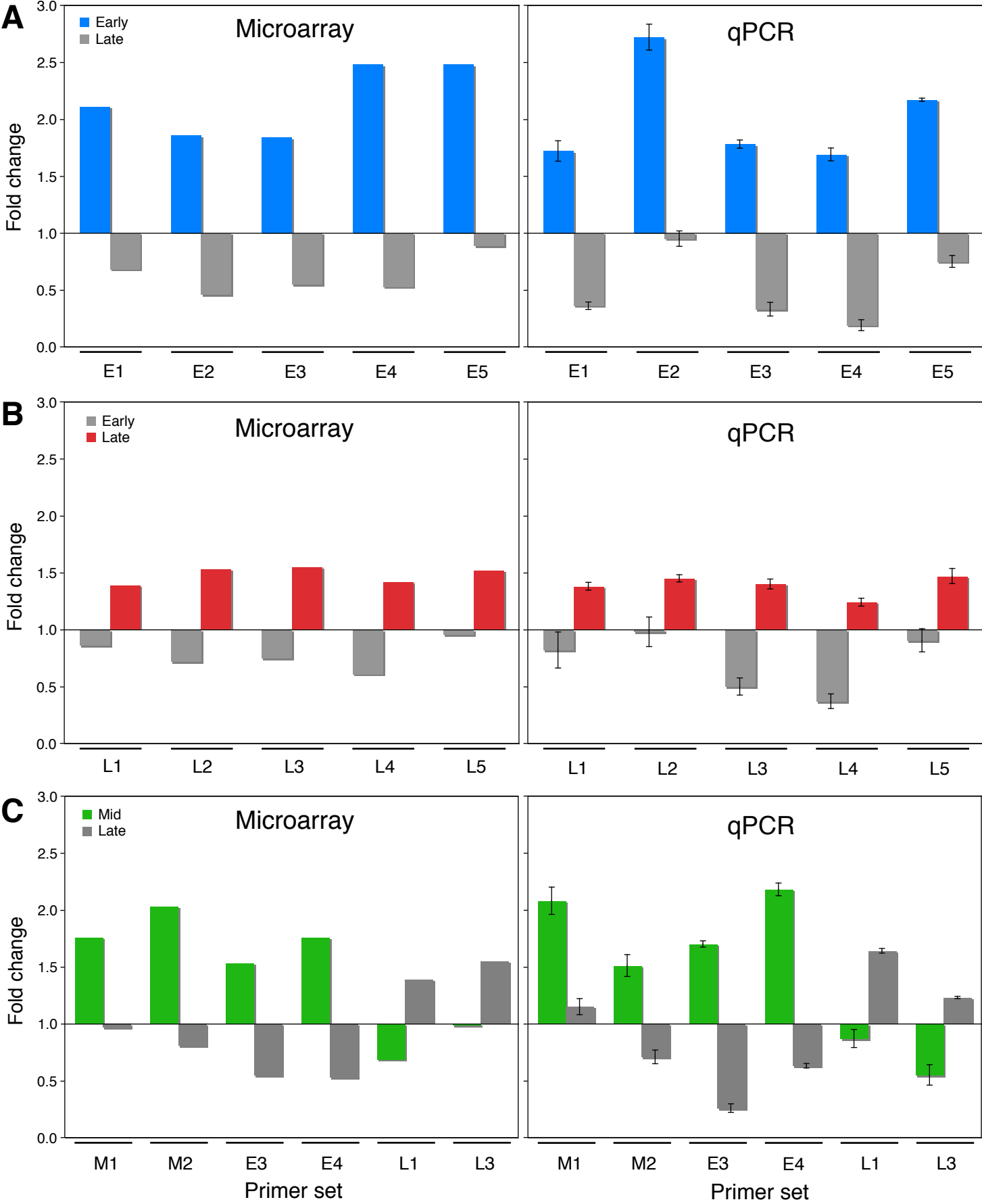

Supplement: Figure S3 — Real time qPCR validation of replication time microarray data. Five primer sets for early and late replicating regions (A,B, respectively) and six for mid replicating regions (C) were used to validate the microarray results (See Table S3 for positions). The barplots show the mean fold change with error bars for the qPCR data indicating ± SE for the three biological replicates. Each qPCR reaction was repeated twice with unamplified IP DNA from each biological replicate. (0.09 MB PDF) [file pgen.1000982.s003.pdf]
