# Supplementary material for: Arabidopsis thaliana Chromosome 4 Replicates in Two Phases That Correlate with Chromatin State
Source: PLoS Genet. 2010 Jun 10;6(6):e1000982. doi: 10.1371/journal.pgen.1000982 (PMC2883604; doi:10.1371/journal.pgen.1000982)

Figure S4. Distribution of genes with select epigenetic patterns within replicons for the long arm of chr4

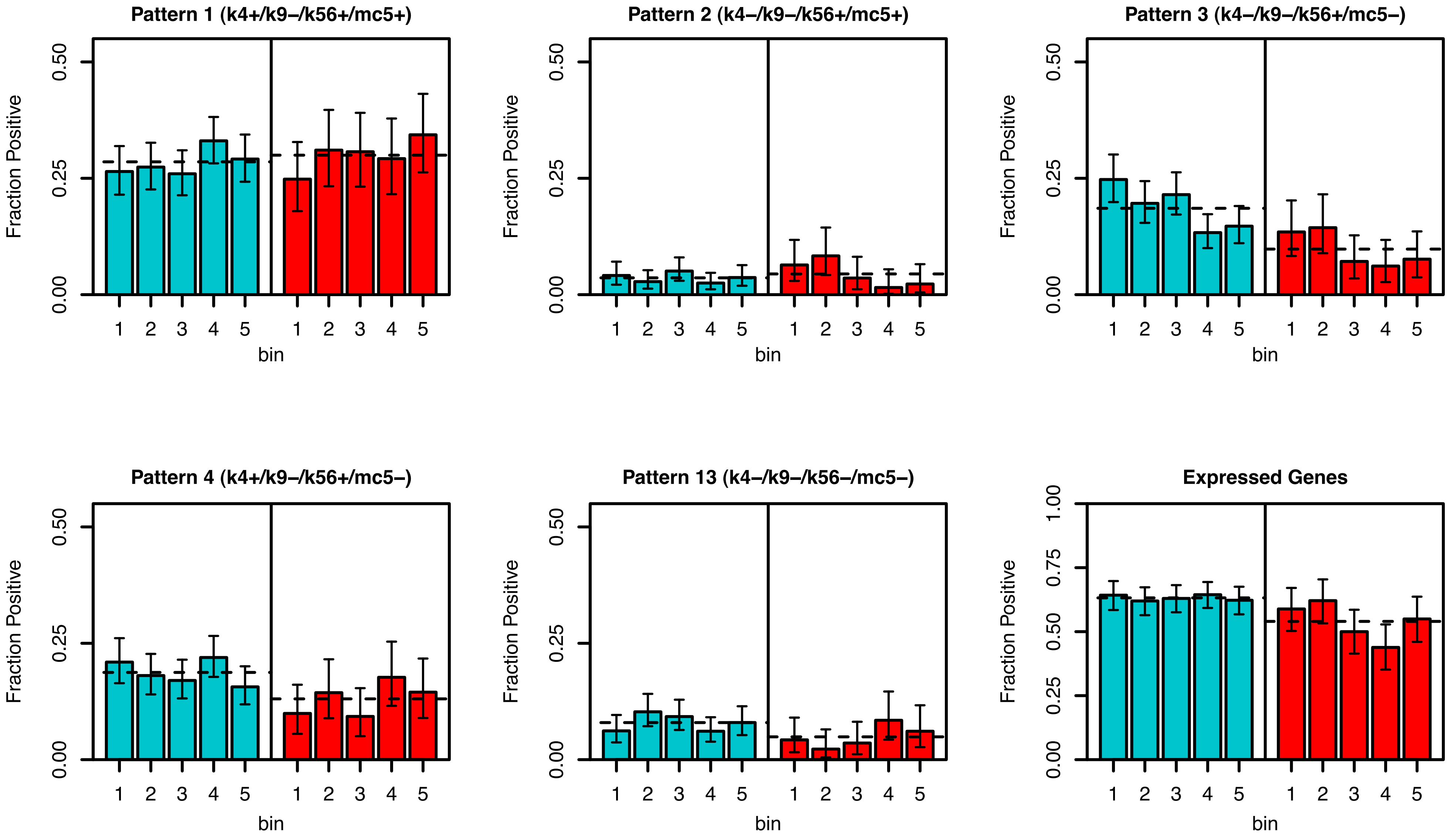

Supplement: Figure S4 — Distribution of genes with select epigenetic patterns within replicons for the long arm of chr4. The epigenetic pattern of chr4 genes was determined from the overlapping probes. Genes with pattern 3 show a slight enrichment near the initiation zones of EM replicons. Genes with patterns 1, 2 and 4 are uniformly distributed across replicons as are all expressed gene, regardless of epigenetic pattern. (0.12 MB PDF) [file pgen.1000982.s004.pdf]
