# Supplementary material for: Arabidopsis thaliana Chromosome 4 Replicates in Two Phases That Correlate with Chromatin State
Source: PLoS Genet. 2010 Jun 10;6(6):e1000982. doi: 10.1371/journal.pgen.1000982 (PMC2883604; doi:10.1371/journal.pgen.1000982)

Figure S5. Distribution of intergenic regions with select epigenetic patterns within replicons for the long arm of chr4

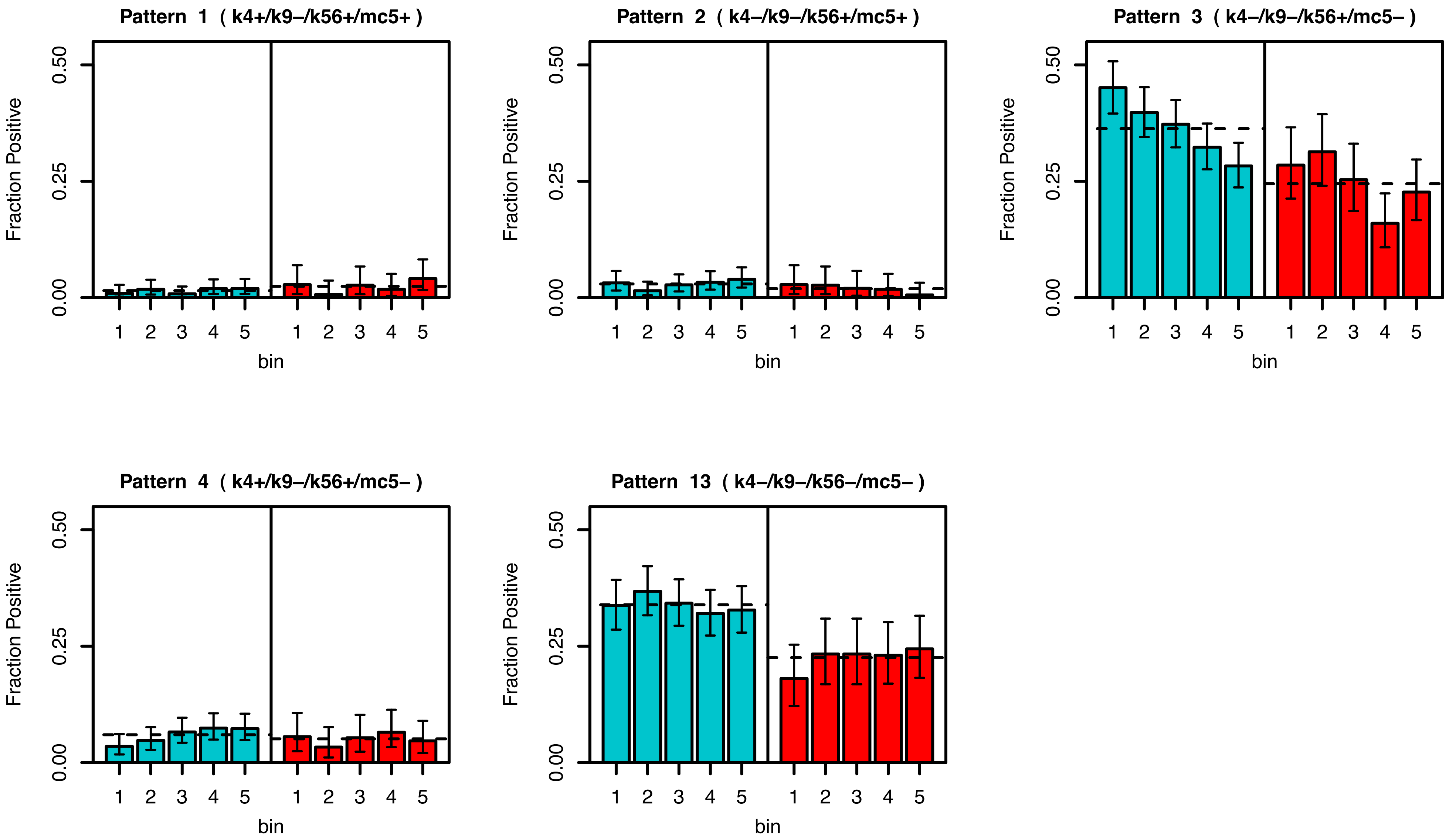

Supplement: Figure S5 — Distribution of intergenic regions with select epigenetic patterns within replicons for the long arm of chr4. Intergenic regions were defined as those regions that did not overlap with any annotated gene. The epigenetic pattern of these regions was determined from the overlapping probes. The two most abundant epigenetic patterns for intergenic regions were patterns 3 and 13. Pattern 3 shows a clear asymmetric distribution across EM replicons whereas regions with pattern 13 are evenly distributed. (0.11 MB PDF) [file pgen.1000982.s005.pdf]
