# Supplementary material for: Arabidopsis thaliana Chromosome 4 Replicates in Two Phases That Correlate with Chromatin State
Source: PLoS Genet. 2010 Jun 10;6(6):e1000982. doi: 10.1371/journal.pgen.1000982 (PMC2883604; doi:10.1371/journal.pgen.1000982)
Supplement: Table S1 — Analysis of S phase nuclei in sorted populations of Arabidopsis suspension cells. (0.04 MB DOC) [file pgen.1000982.s006.doc]

**Table S1. Analysis of S phase nuclei in sorted populations of *Arabidopsis* suspension cells**

|  | Early S/G1 Gate  % S pohas | | | | Mid S Gate | | | | Late S/G2 Gate | | | |
| --- | --- | --- | --- | --- | --- | --- | --- | --- | --- | --- | --- | --- |
|  | Total | BrdU+ | Replicating  (%)^1^ | S phase  (%)^2^ | Total | BrdU+ | Replicating  (%)^1^ | S phase  (%)^2^ | Total | BrdU+ | Replicating  (%)^1^ | S phase  (%)^2^ |
| Experiment 1 | 23010 | 984 | 4.3 | 30.0 | 1475 | 635 | 43.1 | 19.4 | 9206 | 1659 | 18.0 | 50.6 |
| Experiment 2 | 26982 | 1134 | 4.2 | 27.3 | 2021 | 840 | 41.6 | 20.2 | 11723 | 2176 | 18.6 | 52.4 |
| Average |  |  | 4.2 | 28.7 |  |  | 42.3 | 19.8 |  |  | 18.3 | 51.5 |

^1^The percent of BrdU+ nuclei relative to the total population in that gate.

^2^The percent of BrdU+ nuclei relative to the total population of BrdU+ nuclei in all gates.
