# Supplementary material for: Arabidopsis thaliana Chromosome 4 Replicates in Two Phases That Correlate with Chromatin State
Source: PLoS Genet. 2010 Jun 10;6(6):e1000982. doi: 10.1371/journal.pgen.1000982 (PMC2883604; doi:10.1371/journal.pgen.1000982)
Supplement: Table S2 — Reanalysis of sorted populations of nuclei for estimation of purity. (0.06 MB DOC) [file pgen.1000982.s007.doc]

**Table S2.** Reanalysis of sorted populations of nuclei for estimation of purity.

|  | Early nuclei | | Mid nuclei | | Late nuclei | |
| --- | --- | --- | --- | --- | --- | --- |
|  | total | Early S^1^ | total | Mid S^1^ | total | Late S^1^ |
| ***Early S/G1*** |  |  |  |  |  |  |
| Replicate 1 | 18686 | 792 | 578 | 245 | 119 | 22 |
| Replicate 2 | 18397 | 780 | 878 | 371 | 149 | 27 |
| Replicate 3 | 18215 | 772 | 731 | 309 | 477 | 87 |
| Mean (%)^2^ |  | 69.0 |  | 27.6 |  | 4.0 |
| ***Mid S*** |  |  |  |  |  |  |
| Replicate 1 | 1783 | 76 | 14792 | 6258 | 2285 | 418 |
| Replicate 2 | 1560 | 66 | 16402 | 6939 | 1392 | 255 |
| Replicate 3 | 1898 | 80 | 15496 | 6556 | 1945 | 356 |
| Mean (%)^2^ |  | 1.1 |  | 94.0 |  | 4.9 |
| ***Late S/G2*** |  |  |  |  |  |  |
| Replicate 1 | 1020 | 43 | 603 | 255 | 17595 | 3218 |
| Replicate 2 | 464 | 20 | 1492 | 631 | 17591 | 3218 |
| Replicate 3 | 1164 | 49 | 1567 | 663 | 16817 | 3076 |
| Mean (%)^2^ |  | 1.0 |  | 13.9 |  | 85.1 |

**^1^**The number of cells in S phase was estimated using the average percentage of replicating nuclei determined in Table S1.

^2^The mean percent of the specified S phase nuclei relative to the total of all S phase nuclei in that sort.
