# Supplementary material for: Arabidopsis thaliana Chromosome 4 Replicates in Two Phases That Correlate with Chromatin State
Source: PLoS Genet. 2010 Jun 10;6(6):e1000982. doi: 10.1371/journal.pgen.1000982 (PMC2883604; doi:10.1371/journal.pgen.1000982)
Supplement: Table S3 — Sequence and chromosome position of real time qPCR primer sets used for microarray data validation. (0.07 MB DOC) [file pgen.1000982.s008.doc]

**Table S3**. Sequence and chromosome position of real time qPCR primer sets used for microarray data validation

| Replication  timing | Primer | Sequence (5’ → 3’) | Coordinate | |
| --- | --- | --- | --- | --- |
|  |  |  | Start (bp) | End (bp) |
| Early | E1 forward | AAGGGTCTAACTCTCACAAGTACTACG | 723,123 | 724,054 |
|  | E1 reverse | CCTACACGTGTCGACTCTCTAATGATCC |  |  |
|  | E2 forward | TCCGTACGGTCTCGTTTCTTTGCT | 12,373,143 | 12,374,113 |
|  | E2 reverse | GGTTGTGAGTCTTGTCTCTTCTGCCA |  |  |
|  | E3 forward | ACGCATTGCCGCATTGGCTAACAA | 14,353,959 | 14,354,449 |
|  | E3 reverse | AGGCAGACAATGACAGTGATGTGA |  |  |
|  | E4 forward | AACATGCGCCATCCTCTTACTGGA | 17,745,385 | 17,746,223 |
|  | E4 reverse | AGCGCCTGCGAGTGTATTGATAGT |  |  |
|  | E5 forward | ATACGAGGAAGTCAAGCACGTTAAGC | 18,445,846 | 18,446,851 |
|  | E5 reverse | ATTGGTCGCCGTAAGATTTCAAGTTCG |  |  |
| Late | L1 forward | CGCAAAGAGACACGTGCTCCTAGTTA | 4,631,993 | 4,632,771 |
|  | L1 reverse | GCTGCCTCTTTAAAGGTGGTTGGT |  |  |
|  | L2 forward | TTTCTTGGCCTCCCTCCAGTCTTT | 5,961,721 | 5,962,701 |
|  | L2 reverse | ATCTGCATTCAACCAGCAACACCC |  |  |
|  | L3 forward | ATAAGCCTCTGCTTCACCAAGGGA | 9,622,274 | 9,622,788 |
|  | L3 reverse | CGGTTTCTCAGTTGCGCTTGACAT |  |  |
|  | L4 forward | ACCAAGGATCATTTGGAGGTGGCT | 12,202,922 | 12,203,918 |
|  | L4 reverse | AACGATGAGGACGTTGGATACTGG |  |  |
|  | L5 forward | CCAAATTCAGTACCGTAGATTGAGCTGT | 14,481,629 | 14,482,054 |
|  | L5 reverse | ACGTGAACTTCTGGTCGACAGTGA |  |  |
| Mid | M1 forward | ACCTTCAAGGGTTTGAATTTCTTGAGC | 706,389 | 707,350 |
|  | M1 reverse | GTTGGCATTAAACTACAGGTGGTCATC |  |  |
|  | M2 forward | TGTTCACCTGTGGCTATCTTGTATTC | 18,295,361 | 18,296,365 |
|  | M2 reverse | TAGAGCCGACGATGTCATCAATGC |  |  |
| Intermediate | Inter1 forward | TCAACACTCTTAGTGGAAGGTTGACTCT | 12,120,612 | 12,121,166 |
|  | Inter1 reverse | CTGCAGAAGCAAGAGAGTTTGTGAGG |  |  |
|  | Inter2 forward | CAAATGGCAATGCGCAGCTTCAAC | 14,461,366 | 14,462,408 |
|  | Inter2 reverse | TGTCGTTTAGAAAGGCAGGGAGGA |  |  |
