# Supplementary material for: Arabidopsis thaliana Chromosome 4 Replicates in Two Phases That Correlate with Chromatin State
Source: PLoS Genet. 2010 Jun 10;6(6):e1000982. doi: 10.1371/journal.pgen.1000982 (PMC2883604; doi:10.1371/journal.pgen.1000982)
Supplement: Table S4 — Real time qPCR validation of the enriched and depleted regions at different replication timings identified by microarray analysis. (0.06 MB DOC) [file pgen.1000982.s009.doc]

**Table S4**. Real time qPCR validation of the enriched and depleted regions at different replication timings identified by microarray analysis

| Replication  timing | Primer | Coordinate | | Enrichment  by  microarray | Depletion  by  microarray | Amplified IP DNA^1^ | | Unamplified IP DNA^2^ | |
| --- | --- | --- | --- | --- | --- | --- | --- | --- | --- |
|  |  | Start  (bp) | End  (bp) |  |  | Enrichment by qPCR | Depletion by qPCR | Enrichment by qPCR | Depletion by qPCR |
| Early | E1 | 723123 | 724054 | 2.11 | -1.32 | 2.09 | -1.87 | 1.72 | -1.64 |
|  | E2 | 12373143 | 12374113 | 1.86 | -1.54 | 1.92 | -1.69 | 2.72 | -1.05 |
|  | E3 | 14353959 | 14354449 | 1.84 | -1.45 | 1.98 | -2.13 | 1.78 | -1.67 |
|  | E4 | 17745385 | 17746223 | 2.48 | -1.47 | 2.19 | -1.59 | 1.69 | -1.81 |
|  | E5 | 18445846 | 18446851 | 2.48 | -1.11 | 2.70 | 1.01 | 2.17 | -1.25 |
| Late | L1 | 4631993 | 4632771 | 1.39 | -1.14 | 1.22 | -1.48 | -1.18 | 1.38 |
|  | L2 | 5961721 | 5962701 | 1.53 | -1.28 | 1.80 | -1.61 | -1.02 | 1.45 |
|  | L3 | 9622274 | 9622788 | 1.55 | -1.25 | 2.09 | -1.31 | -1.50 | 1.40 |
|  | L4 | 12202922 | 12203918 | 1.42 | -1.39 | 1.23 | -1.53 | -1.63 | 1.24 |
|  | L5 | 14481629 | 14482054 | 1.52 | -1.04 | 2.02 | 1.12 | -1.09 | 1.47 |
| Intermediate | Inter1 | 12120612 | 12121166 | 0.97 | -1.08 | -1.06 | -1.16 | 1.00 | 1.00 |
| Intermediate | Inter2 | 14461366 | 14462408 | 1.07 | 1.00 | 1.01 | -1.21 | 1.00 | 1.00 |
| Mid | M1 | 706389 | 707350 | 1.76 | -1.03 | 2.27 | 1.53 | 2.08 | 1.15 |
|  | M2 | 18295361 | 18296365 | 2.03 | -1.19 | 1.79 | -1.02 | 1.51 | -1.29 |
|  | E3 | 14353959 | 14354449 | 1.53 | -1.45 | 1.58 | -1.03 | 1.70 | -1.74 |
|  | E4 | 17745385 | 17746223 | 1.76 | -1.47 | 2.44 | -1.04 | 2.18 | -1.37 |
|  | L1 | 4631993 | 4632771 | 1.39 | -1.31 | 1.20 | -1.49 | -1.13 | 1.64 |
|  | L3 | 9622274 | 9622788 | 1.55 | -1.01 | 1.54 | -1.04 | -1.45 | 1.23 |
| Intermediate | Inter2 | 14461366 | 14462408 | 1.12 | 1.00 | 1.24 | 1.45 | 1.00 | 1.00 |

^1^ Total 50 pg of amplified IP DNA was used as a template DNA with all primer sets.

^2^ Inter2 primer set was used to normalize enrichment and depletion of unamplified IP DNA, which is single strand DNA, with all other

primer sets.
