# Supplementary material for: Arabidopsis thaliana Chromosome 4 Replicates in Two Phases That Correlate with Chromatin State
Source: PLoS Genet. 2010 Jun 10;6(6):e1000982. doi: 10.1371/journal.pgen.1000982 (PMC2883604; doi:10.1371/journal.pgen.1000982)
Supplement: Text S1 — BrdU and qPCR materials and methods. (0.05 MB DOC) [file pgen.1000982.s018.doc]

**Supplemental Materials and Methods**

***BrdU dot blot assay*** (Figure S1 panel C)

BrdU pulse-labeled genomic DNA extracted from cell samples was denatured at 95°C for 5 min, followed by an immediate cooling on ice. One μl of TE buffer containing 25 ng of DNA was spotted on a Durolose (Stratagene) membrane and dried in the dark for 10 min. The membrane was UV crosslinked and blocked using Odyssey blocking buffer (LI-COR biosciences) for 1 hr with gentle agitation. An anti-BrdU antibody (Invitrogen) was added at 1:10,000 dilution, and the incubation was continued overnight at 4°C with gentle agitation. The membrane was then washed for 10 min in TBST buffer (pH8, 0.1% Tween 20) 3 times and incubated for 2 hrs in Odyssey blocking buffer containing a 1:10,000 anti-mouse Alexa 688 (Invitrogen) with gentle agitation, followed by three 10 min washes in TBST buffer and subsequently washed briefy with sterile water. The BrdU-labeled DNA was quantified by scanning the membrane at channel 700 with an Odyssey scanner (LI-COR biosciences).

## Real time quantitative PCR

Real time quantitative PCR was carried out with a Stratagene Mx3000P Real-Time PCR System. All qPCR reactions were performed using 300 nM primers and Tm = 55°C using Perfecta SYBR Green SuperMix (Quanta Biosciences). Primer pairs were designed by PrimerQuest (Intergrated DNA Technologies), with final PCR products ranging in size from 150 to 200 bp. A list of primer pairs and their detailed information is included in Tables S1 and S2.
